# Autophagy Blockade by Ai Du Qing Formula Promotes Chemosensitivity of Breast Cancer Stem Cells Via GRP78/β-Catenin/ABCG2 Axis

**DOI:** 10.3389/fphar.2021.659297

**Published:** 2021-06-03

**Authors:** Mianmian Liao, Caiwei Wang, Bowen Yang, Danping Huang, Yifeng Zheng, Shengqi Wang, Xuan Wang, Juping Zhang, Chunbian Tang, Zheng Xu, Yu He, Ruolin Huang, Fengxue Zhang, Zhiyu Wang, Neng Wang

**Affiliations:** ^1^ The Research Center for Integrative Medicine, School of Basic Medical Sciences, Guangzhou University of Chinese Medicine, Guangzhou, China; ^2^ Department of Medical Biotechnology, School of Basic Medical Sciences, Guangzhou University of Chinese Medicine, Guangzhou, China; ^3^ Integrative Research Laboratory of Breast Cancer, The Second Clinical College, Guangzhou University of Chinese Medicine, Guangzhou, China; ^4^ Guangdong Provincial Key Laboratory of Clinical Research on Traditional Chinese Medicine Syndrome, Guangdong Provincial Academy of Chinese Medical Sciences, Guangdong Provincial Hospital of Chinese Medicine, Guangzhou, China; ^5^ Shenzhen Clinical Medical College, Guangzhou University of Chinese Medicine, Guangzhou, China; ^6^ Department of Hepatology, Shenzhen Traditional Chinese Medicine Hospital, The Fourth Clinical Medical College of Guangzhou University of Chinese Medicine, Shenzhen, China

**Keywords:** breast cancer chemosensitivity, cancer stem cells, autophagy, Ai Du Qing formula, GRP78/β-catenin/ABCG2 axis

## Abstract

Accumulating evidence suggests that the root of drug chemoresistance in breast cancer is tightly associated with subpopulations of cancer stem cells (CSCs), whose activation is largely dependent on taxol-promoting autophagy. Our pilot study identified GRP78 as a specific marker for chemoresistance potential of breast CSCs by regulating Wnt/β-catenin signaling. Ai Du Qing (ADQ) is a traditional Chinese medicine formula that has been utilized in the treatment cancer, particularly during the consolidation phase. In the present study, we investigated the regulatory effects and molecular mechanisms of ADQ in promoting autophagy-related breast cancer chemosensitivity. ADQ with taxol decreasing the cell proliferation and colony formation of breast cancer cells, which was accompanied by suppressed breast CSC ratio, limited self-renewal capability, as well as attenuated multi-differentiation. Furthermore, autophagy in ADQ-treated breast CSCs was blocked by taxol *via* regulation of β-catenin/ABCG2 signaling. We also validated that autophagy suppression and chemosensitizing activity of this formula was GRP78-dependent. In addition, GRP78 overexpression promoted autophagy-inducing chemoresistance in breast cancer cells by stabilizing β-catenin, while ADQ treatment downregulated GRP78, activated the Akt/GSK3β-mediated proteasome degradation of β-catenin *via* ubiquitination activation, and consequently attenuated the chemoresistance-promoted effect of GRP78. In addition, both mouse breast cancer xenograft and zebrafish xenotransplantation models demonstrated that ADQ inhibited mammary tumor growth, and the breast CSC subpopulation showed obscure adverse effects. Collectively, this study not only reveals the chemosensitizating mechanism of ADQ in breast CSCs, but also highlights the importance of GRP78 in mediating autophagy-promoting drug resistance *via* β-catenin/ABCG2 signaling.

## Introduction

Breast cancer remains the most common malignant cancer among women, and is the second most common cause of death in women, with 8.6 million new cases and 4.2 million related deaths in 2018 (Bray et al., 2018; Byrd et al., 2020; Shieh and Tice, 2020). To improve the overall survival and life quality of patients with breast cancer, multiple strategies have arisen, including surgery, radiotherapy, endocrine, targeted therapy, and chemotherapy (Britt et al., 2020). However, the benefits of chemotherapy are largely limited by tumor-promoting host responses and non-selective toxicity (Rizzo, 2020). Chemotherapy involves the generating of pro-metastatic signals, eliciting primary breast cancer cells transferring and seeding into distant pulmonary organs (Minami et al., 2016). TAOK3 is a candidate protein target in breast cancer chemoresistance, which possibly acts by interacting with NF-κB, as revealed by kinase short hairpin RNA (shRNA) screening (Lai et al., 2020). KCNN4 promoted gemcitabine resistance in breast cancer models by activating RAS-MAPK and PI3K-AKT signaling and subsequently upregulating of BCL2A1 protein levels, and its expression predicted poor disease-free survival (Lin et al., 2020). In addition, resistance to the anti-microtubule inhibitor eribulin eventually developed in more than 90% breast cancer patients, despite initial exciting therapeutic effects. Taken together, searching for a novel chemosensitizing therapy may have profound implications for the control and prevention of breast cancer recurrence.

Cancer stem cells (CSCs), also known as tumor-initiating cells, belong to a small population of cancer cells characterized by stem-cell properties, such as a self-renewal ability, multi-directional differentiation, tumorigenic potential, pro-metastatic inclination, and resistance to chemotherapy (Bai et al., 2018; Sharif et al., 2019; Mohiuddin et al., 2020). CSCs have always been associated with tumor relapse in various tumors, including breast cancer, lymphoma, liver cancer, esophageal cancer and others (Nguyen et al., 2012; Song et al., 2020). CD44^high^NRF2^high^ subpopulations have distinct CSC-like capabilities of accelerating tumor proliferation, enhancing sphere formation and resistance to anticancer drugs in breast cancer (Ryoo et al., 2018). The reprogramming of cancer-associated fibroblasts is triggered by Hedgehog signaling, subsequently leading to chemoresistance, a CSC-like phenotype, as well as enhanced fibrillar collagen in triple-negative breast cancer (Cazet et al., 2018). The up-regulated gene *TSPAN8* in breast CSCs interacts with PTCH1 and restrains the degradation of SHH/PTCH1 compounds by recruiting the deubiquitinating enzyme ATXN3, thereby promoting the expression of stem genes *NANOG*, *OCT4*, as well as *ALDHA1*, which results in tumor formation, chemoresistance, and poor prognosis (Zhu et al., 2019). The proportion of CD44^+^/CD24^−/low^ after chemotherapy therapy was 9.5 times higher than that before chemotherapy in breast cancer, and breast CSCs served as an independent risk factor for predicting poor survival in breast cancer patients (Tanei et al., 2009).

Accumulating evidence has shown that the Wnt/β-catenin pathway not only determines mammary development, but also regulates self-renewal, tumorigenesis and differentiation of CSCs in various cancers including breast cancer, lung cancer, and prostate cancer. β-catenin is the core target of this signaling, and its nuclear accumulation and translocation leads to transcription of downstream genes such as *ABCG2*, *HMGB1*, *cyclin D1*, *c-myc*, and *Axin2*, which are strongly correlated to cancer chemosensitivity (Cheng et al., 2019; Rahmani et al., 2020; Yousefnia et al., 2020). Upon the activation of β-catenin, the complex composed of Axin and glycogen synthase kinase-3β (GSK-3β) prevents the phosphorylation and degradation of β-catenin, increases the concentration of β-catenin in cytoplasm, and then transfers into the nucleus to interact with T cell transcription factor/lymphoid enhancer factor to activate the Wnt1/β-catenin signal pathway, and finally start the transcription of downstream target genes such as *cyclin D1* and *c-myc*, resulting in abnormal proliferation of CSCs and tumor drug resistance (Maiese, 2014; Schuijers et al., 2014; Ashouri et al., 2016; Hassanian et al., 2016). Silencing MALAT1 significantly reduced CD133^+^CD90^+^ stem cell proportions, attenuated sphere formation abilities as well as the survival and differentiation potential of CSCs mainly by downregulating β-catenin expression (Chang et al., 2020). More importantly, GRP78, an endoplasmic reticulum chaperone, is a novel target that closely associated with breast CSC resistance by regulating β-catenin/ABCG2 signaling (Wang N. et al., 2014), which highlights the significance of GRP78 in mediating drug resistance and β-catenin signaling in CSCs. However, the specific regulatory mechanism of GRP78 in mediating cancer resistance and β-catenin signaling in CSCs remains obscure.

Emerging evidence has revealed that autophagy is crucial in different stages of tumor development and plays a central role in the survival, self-renewal and differentiation of CSCs, which has been tightly implicated in resistance to chemotherapy (Ding and Hong, 2020; Yamamoto et al., 2020). Autophagy, which is regarded as one of the evolutionarily conserved degradation mechanisms of living organisms, is an adaptive catabolic process that is responsible for maintaining cellular bioenergetics, clearing aggregated proteins, and degrading damaged organelles and macromolecules under the regulation of autophagy-related genes (Galluzzi and Green, 2019). Autophagy maintains the expression of breast CSC CD44^+^/CD24^−/low^ fractions by secreting CD24 and IL-6 in breast cancer (Maycotte et al., 2015). Inhibition of autophagy regulatory factor FIP200 reduces the tumorigenesis-promoting ability of breast CSCs *via* STAT3 and TGFβ/Smad pathways (Yeo et al., 2016). GRP78 cooperates with KSR2 to promote ER translocation *via* the MAPK pathway in mutant melanoma cells to mediate autophagy, thereby resulting in chemotherapy resistance (Ojha et al., 2019). Additionally, miR-495-3p targets GRP78 and regulates mammalian target of rapamycin (mTOR) to modulate autophagy in MDR cells, thereby sensitizing gastric cancer cells to chemotherapy (Chen et al., 2018). Furthermore, GRP78 mediates autophagy, leading to the resistance of melanoma cells to temozolomide (Ryabaya et al., 2018). To date, multiple autophagy-inhibiting drugs are being tested in clinical trials for anti-cancer treatment, but only two have been approved by the FDA to regulate autophagy activity in clinic applications, i.e., chloroquine (CQ) and hydroxychloroquine (HCQ) (Sharma et al., 2019; Chang et al., 2020). Although HCQ is currently being investigated in different phases of clinical trials for anti-oncology, its cumulative toxic effects greatly limit the therapeutic range of anti-cancer drugs (Limpert et al., 2018). Therefore, it is essential to investigate whether GRP78 could regulate autophagic homeostasis in breast CSC chemoresistance to identify for novel autophagic targets and corresponding inhibitors for further clinical application.

Traditional Chinese medicine (TCM) has historically been used in treatment and prevention of cancer because of its multi-target, and multi-substance characteristics, as well as excellent safety profile (Ma et al., 2019; Liu et al., 2020). ADQ formula, which is composed of four Chinese herbs, namely, *Hedyotis diffusa*, *Curcuma zedoaria*, *Astragalus membranaceus* and *Glycyrrhiza uralensis Fisch*, has shown sound clinical efficacy in prolonging the survival, reducing side effects, as well as improving the quality of life of cancer patients. Each herb in ADQ could restrict cancer growth through cell cycle arrest, apoptosis induction, as well as immunomodulation. The ethanol extract of *Hedyotis diffusa* could significantly limit the proliferation and induce apoptosis of highly metastatic breast cancer cells MDA-MB-231 and MDA-MB-453, but had no obvious cytotoxic effect on a variety of non-malignant cells (Yang et al., 2019). *Hedyotis diffusa polysaccharide* inhibited cell adhesion, invasion and migration of human lung adenocarcinoma A549 cells by downregulating matrix metalloproteinase (Lin et al., 2019). Tulip improves the sensitivity of tumor-resistant cells to doxorubicin and docetaxel by downregulating the expression of P-gp and inducing S-phase arrest (Yang et al., 2011). Astragaloside IV from *Astragalus membranaceus* enhances the chemo-sensitivity of taxol to breast cancer by targeting oxidant damage through Caveolin-1 (Zheng et al., 2019). Isoliquiritigenin extracted from *Glycyrrhiza*, targets miR-25 to regulate autophagy and thus increase chemosensitivity in breast cancer (Wang Z. et al., 2014). Studies have also confirmed that liquiritigenin extracted from licorice root apparently suppresses the invasion and epithelial-mesenchymal transition of colorectal cancer by downregulating the expression of Runx2 and inactivating the PI3K/AKT signaling pathway (Meng and Lin, 2019). More importantly, our pilot research illustrated that ADQ suppressed the proliferation of parental and chemoresistant breast cancer cells by promoting G2/M blockage and apoptosis induction by taxol, thereby enhancing the sensitivity of breast cancer to taxol *in vivo* and *in vitro* (Wang et al., 2018). GRP78 has also been found to be one of the core targets of ADQ, while the effect of GRP78 in influencing ADQ-mediated breast cancer chemosensitization was not assessed. Overall, elucidating the underlying molecular mechanism by which ADQ enhances the sensitivity of breast cancer to chemotherapeutic drugs can improve the clinical outcomes of cancer patients.

In this study, we show that ADQ inhibits the proliferation of breast cancer and enhances the chemosensitivity of taxol to breast cancer. ADQ also limits the self-renewal, differentiation ability and autophagy activity of breast CSCs. Further investigation demonstrated that ADQ could mediate autophagy *via* GRP78/β-catenin/ABCG2 signaling, thereby inhibiting the resistance of breast CSCs to taxol. *In vivo* studies have also confirmed that the synergistic effect of ADQ and taxol restricts the growth of breast cancer by downregulating GRP78, β-catenin and LC3 expression. Our study provides experimental basis for the clinical application of ADQ, as well as highlights the novel role of GRP78 in regulating autophagy, thus establishing the role of GRP78 in regulating β-catenin signal transduction of breast CSC chemoresistance.

## Materials and Methods

### Preparation of Ai Du Qing

Ai Du Qing formula (ADQ) was derived from four herbs, the dried whole plants of *Scleromitrion diffusum* (Willd.) R. J. Wang [Rubiaceae] (15 g) (Barcode:2706553), rhizome of *Curcuma aromatica* Salisb. [Zingiberaceae] (15 g) (Barcode:320230), root of *Astragalus mongholicus Bunge* [Fabaceae] (15 g) (Barcode:1261257) and root and rhizome of *Glycyrrhiza uralensis* Fisch. ex DC. [Fabaceae] (15 g) (Barcode:11895)*,* which were purchased from the pharmacy of Guangdong Provincial Hospital of Traditional Chinese Medicine. For drug preparation, each herb of ADQ were mixed at a 1:1:1:1 ratio and then underwent mechanical trituration into a fine powder (180 g), efflux extraction for 1 h thrice with 2 L ethanol, inspissation, and lyophilization to a final production ratio of 7.2–9.6%. For quality control analysis, an Agilent 1,260 system combined with diode array detection (DAD) (Agilent, Palo Alto, CA, United States) and an Agilent C_18_ column (5 μm, 250 mm × 4.6 mm) with a HPLC guard cartridge system (Phenomenex, SecurityGuard) were applied for HPLC analysis. The mobile phases were composed of acetonitrile (A) and 0.05% (v/v) phosphoric acid (B) using a gradient program of 15% A in 0–23 min, 15–38% A in 23–40 min, 38% A in 40–50 min, 38–60% A in 50–60 min, 60% in 60–75 min. The flow rate was 1.0 ml/min, the column temperature was set to 30°C, and the DAD detector was set at 216, 236, 260, 276, and 308 nm. P-coumaric acid, calycosin-7-glucoside, liquiritin, glycyrrhizic acid, and curcumol were prepared and diluted with methanol for the preparation of standard solutions. A total of 10 µL of these solutions were injected for HPLC analysis and the calibration curves were constructed by plotting the peak areas of the analysis. ADQ (0.1 g) was dissolved in 20 mL methanol to prepare the sample solutions. Then extracting solution was filtrated through 0.2 µm membrane filter for HPLC analysis after sonicating for 60 min. The procedures of drug preparation and quality control were conducted according to our pilot study (Wang et al., 2018).

### Cell Culture

The human breast cancer cell lines MCF-7 and MDA-MB-231, human mammary epithelial cells with integrated SV40 gene (HBL-100) as well as non-malignant mammary epithelial cell line MCF-10A were obtained from ATCC (Manassas, VA, United States). All the above cell lines have been identified by short tandem repeat analysis. RPMI-1640 medium (Gibco Life Technologies, Lofer, Austria) for MCF-7 cells and Dulbecco's Modified Eagle medium (DMEM, Gibco Life Technologies, Lofer, Austria) for MDA-MB-231 cells and HBL-100 cells were applied in exception to 10% fetal bovine serum (Gibco) and 1% penicillin and streptomycin (Gibco). And MCF-10A cells were maintained in DMEM/F12 medium (Gibco) supplemented with 5% horse serum (HyClone, Logan, Utah, United States), 1% penicillin and streptomycin, 20 ng/ml recombinant human epidermal growth factor (BD Bioscience, Bedford, MA, United States), 0.5 μg/ml hydrocortisone (STEMCELL Technologies, Vancouver, Canada), 100 ng/ml cholera toxin (MACGENE, Beijing, China) and 10 μg/ml insulin (Sigma, St. Louis, MO, United States). DMEM/F12 medium for the CSCs derived from MDA-MB-231 and MCF-7 cells were employed in addition to B27 (Invitrogen, Carlsbad, CA, United States), 5 μg/ml of insulin, 20 ng/ml of hEGF, 1% penicillin and streptomycin and 0.4% BSA (Sigma) at 37°C with 5% CO_2_.

### Cell Viability Detection and Colony Formation Assays

The sensitivity and cytotoxicity of cells to ADQ and taxol were analyzed with CCK-8 kit (KeyGEN Bio TECH, Nanjing, China). Briefly, 3 × 10^3^ cells per well were seeded in 96-well microplates and incubated overnight. After cells attachment, taxol (Bristol-Myers Squibb Company, Princeton, NJ) or ADQ formula dissolved in DMSO were added to the wells for 24, 48 or 72 h, respectively. The Graphpad Prism software was administered to calculate the IC_50_ inhibitory drug concentration and plot the curves of cell proliferation. A triplicate independent experiment was performed. For cell counting, 3 × 10^5^ cells per well were planted into 6-well plates and allowed to adhere to the bottom of the plate overnight. After adding the corresponding therapeutic drugs, cell numbers were calculated using trypan blue exclusion on a Cellometer Mini device (Nexcelom, Boston, MA, United States). A triplicate independent experiment was performed. For colony formation assay, 1 × 10^3^ cells per well were plated into the 6-well plate overnight to gain further insights into the combination of ADQ formula and taxol. Next, the adherent wells were exposed to ADQ formula or taxol alone or in combination for 4 h and then cultured with fresh medium for two weeks. The colonies fixed with 4% paraformaldehyde were dyed with 0.5% Crystal violet (Beyotime Biotechnology, Shanghai, China), and then scanned and counted under the microscope. A triplicate independent experiment was performed.

### Flow Cytometry Analysis

For the drug efflux assay, 3 × 10^5^ cells per well were cultured on 6-well plates and then pretreated with 50 μg/ml ADQ formula after adherence. After 48 h, the drug-containing medium was changed with fresh complete medium containing 10 μg/ml epirubicin (Selleck, Shanghai, China) for 24 h at 37°C. Then the cells were washed, harvested with phosphate-buffered saline (PBS, Gibco) for flow cytometry analysis with BD LSRFortessa (BD Biosciences, San Diego, CA, United States) and analyzed using FlowJo cytometry analysis software. A triplicate independent experiment was performed. For CSC ratio detection, 3 × 10^5^ cells per well were cultivated into 6-well plates overnight and then pretreated alone or in combination with 50 μg/ml ADQ formula or 50 nM taxol. After 48 h, the cells were digested with 0.25% trypsin (Gibco), rinsed once with PBS and resuspended in the wash buffer (10^6^ cells/100 μl) containing 2% FBS. Then the cell suspension was dyed with 5 μl CD44-FITC, 5 μl CD24-PE (BD Biosciences) and FITC-and PE-labeled isotype IgG1 served as the negative control. The cells were subsequently incubated at 4°C in the dark for 40 min and then washed three times with PBS containing 2% FBS. BD LSRFortessa was applied to analyze the processed samples. The results were analyzed by FlowJo software. A triplicate independent experiment was performed. For the identification and evaluation of breast CSCs, the ALDEFLUOR™ Kit (STEMCELL Technologies) was used to analyze primary breast cancer cells derived from BALB/C-nude mice. Briefly, mice mammary tumors were made into a single-cell suspension (1 × 10^6^ cells per ml), and resuspended into 500 μl ALDEFLUORTM assay buffer. Then, dividing the cell suspension into two parts, one part was incubated with ALDEFLUORTM Diethylaminobenzaldehyde (DEAB) Reagent, a specific inhibitor of ALDH activity, which was utilized to regulate background fluorescence in the ALDH staining assay, the other part was hatched with the activated ALDEFLUORTM Reagent at 37°C for 40 min to label ALDH^+^ cells, which were considered as breast CSCs. Lastly, the cells were analyzed with the BD LSRFortessa flow cytometer after washed with PBS for three times. The gating strategy of flow cytometry was according to the instructions of *A Guide To ALDH* ^*br*^ *Cell Flow Cytometry Setup* (STEMCELL, Catalog #01700). A detailed illustration is given in Supplementary Figure S1. FACS gating strategy was utilized for the analysis of live ALDH^+^ subsets in mice. A cell sample exposed to ALDEFLUOR™ in the presence of the ALDH inhibitor, DEAB, is the only appropriate negative control for this assay. First plot gating (FSC-A, SSC-A subset) for live cells, then second (FSC-H, FSC-W subset) and third plot (SSC-H, SSC-W subset) for single cells by excluding RBCs and debris. Create a FITC vs. SSC dot plot, gated on R1. Adjust the FITC photo-multiplier tube voltage, and line the rightmost edge of the stained DEAB control population with the second log decade on the FITC axis. Then remove the DEAB tube from the cytometer, and place the corresponding SAMPLE tube into the cytometer. Gate on the ALDH^+^ population (R2). Remove the SAMPLE tube. Cells with high ALDH activity can only be identified in comparison with the background fluorescence levels of the DEAB control sample (R2-R1). A triplicate independent experiment was performed.

### Cancer Stem Cells Spheres Formation

The cancer stem cell culture medium, consisted of DMEM/F12 medium supplemented with B27, 20 ng/ml hEGF, 5 μg/ml insulin, 0.4% BSA and 1% penicillin and streptomycin, was used to selectively cultivate breast CSCs sorted from MDA-MB-231 and MCF-7 cells. And then they were seeded and cultured in ultralow adhesion 6-well plates at a density of 3 × 10^5^ cells per well. To investigate the impact of ADQ formula on the formation of CSC spheres, ADQ formula was added to each well with or without taxol. The complete medium was replaced every two days using a half-change method without other processing and the size as well as numbers of primary spheres were assessed under a microscope every day. To evaluate the relative numbers of CSCs spheres for the second generation, the primary spheres were gathered and blow to single-cell suspensions, and then replated in ultralow attachment plates without other treatment. The sizes of the CSCs sphere were observed using the above method. A triplicate independent experiment was performed.

### Cancer Stem Cells Differentiation

For the differentiation assay, the 8th day-CSCs spheres of MDA-MB-231 and MCF-7 cells were loaded into 6-well plates and cultivated in RPMI-1640 complete medium or DMEM complete medium supplemented with 10% fetal bovine serum and 1% penicillin and streptomycin. Then morphological changes of CSCs spheres were observed with the microscope for 0, 1, 3, 6, 12, 24 h. A triplicate independent experiment was performed.

### Western Blotting Analysis

The cells treated with ADQ and taxol were lysed using radio immunoprecipitation assay (RIPA) buffer (Beyotime) in the presence of a protease inhibitor mixture (Roche Diagnostics, IN) on ice. The extraction of nuclear protein and cytoplasmic protein were extracted according to the instructions of the ProtLytic Nuclear and Cytoplasmic Protein Extraction kit (NCM Biotech). The bicinchoninic acid assay (Thermo Fisher Scientific, Bonn, Germany) was applied to quantify concentration of protein solutions. Protein lysates (60 μg) were resolved on 10% or 12% sodium dodecyl sulfate polyacrylamide gel electrophoresis (SDS-PAGE) according to the molecular weight of the protein and subsequently transferred onto a polyvinylidene fluoride microporous membrane (PVDF, GE Healthcare, Freiburg, Germany). Blocked membranes with 5% milk were incubated with primary antibodies, including β-catenin (no.66379-1-lg), phosphorylated β-catenin (*P*-β-catenin, no.80067-1-RR), GRP78 (no. 11587-1-AP), LC3 (no. 14600-1-AP), AKT (no. 60203-2-lg) from Proteintech (Rosemont, United States); P62 (no. 18420-1-AP), ABCG2 (no. BF0058), phosphorylated AKT (*P*-AKT, no. 58169S), GSK-3β (no. AF5016), phosphorylated GSK-3β (*P*-GSK-3β, no. AF 2016) from Affinity (Cincinnati, United States); β-actin(no. 58169S) from CST (Boston, MA, United States) overnight at 4°C. Then the membrane was rinsed with TBST three times and incubated with corresponding secondary anti-rabbit or anti-mouse from Proteintech for 2 h at room temperature. The protein bands were visualized using the enhanced chemiluminescence detection reagents (Tanon, Shanghai, China) and quantified digitized by ImageJ software. A triplicate independent experiment was performed.

### LC3-mRFP-GFP Lentiviral Transfection

To construct autophagy LC3 dual-labeled lentivirus stable cell lines, breast cancer cells MDA-MB-231, MDA-MB-231-rGRP78 and MDA-MB-231-shGRP78 were exposed to AVV-mRFP-GFP-LC3 lentivirus. Specifically, 1 × 10^5^ cells per well were cultivated in 24-well plates overnight and then infected with AVV-mRFP-GFP-LC3 lentiviral vectors (HanBio Technology, Shanghai, China) for 48 h according to the manufacturer's instructions. The lentivirus-containing medium was replaced with fresh RPMI-1640 or DMEM complete medium supplemented with 10% fetal bovine serum and 1% penicillin and streptomycin subsequently, and puromycin was added to construct stable cell lines from 0.1 μg until the cells were not killed.

### Immunofluorescence Analysis

With regard to the expression and distribution of β-catenin, 1 × 10^5^ cells per well were planted on cover slips inside the 24-well culture plates overnight. After the indicated treatment, cells washed with PBS were fixed with 4% paraformaldehyde (PFA, NCM Biotech, Suzhou, China) for 30 min and permeabilized with 0.05% triton X-100 (Sigma) for 15 min, and followed by goat serum (Beyotime) blocking for 60 min. Following incubating with primary antibody of β-catenin at 4°C overnight, the samples were subsequently labeled with an anti-mouse IgG conjugated with Alexa Fluor^®^ 555 (Thermo scientific, Waltham, MA, United States) for 1 h at room temperature in the dark. Cell nucleus was finally stained with 4′,6-diamidino-2-phenylindole (DAPI, Sigma-Aldrich) for 10 min and LMS710 confocal microscope (ZEISS, Jena, Germany) was applied to capture the fluorescence signals. To study the effect of ADQ on delivery of cargo to lysosomes, stable breast CSCs lines infected with lentivirus were planted at a density of 2.5 × 10^5^ cells per well in laser confocal dishes overnight. After the indicated drug for 48 h, the fluorescence images were detected by LMS710 confocal microscope and the fluorescence intensity was measured by ImageJ software. For Lysosome marker detection, cells administered with ADQ formula or taxol for 48 h, were exposed to 10 μg/ml DQ-BSA (Thermo Fisher Scientific, waltham, United States) for 24 h followed by incubated with 1 μg/ml Lysored (KeyGEN BioTECH, Nanjing, China) for 15 min at 37 °C. Then DAPI was applied to nuclear staining for 10 min. The fluorescence signals were observed through LMS710 confocal microscope and digitized by ImageJ software. The mammospheres were dissociated into single-cell suspension for quantification of autophagosome/autolysosome under a higher magnification.

### Transfection of Plasmid and Small Interfering RNA

The commercialized recombinant plasmids for GRP78 and the scrambled plasmids were obtained from Vigene Biosciences (Jinan, China). MCF-7 and MDA-MB-231 cells were transfected with plasmids using LipoFiter™ reagent (Hanbio Biotechnology Co., LTD. Shanghai, China) according to the manufacturer’s instructions. After 24 h, the transfected cells were passaged and selected for two weeks with 5 μg/ml puromycin (Invitrogen, Carlsbad, United States) or 400 μg/ml G418 (Invitrogen) to construct GRP78 stable cell lines. Negative control cell lines were generated by transfecting cells with scrambled plasmids. The pcDNA 3.1(+)-β-catenin plasmid and the siRNA targeting β-catenin purchased from Invitrogen, were transfected into MDA-MB-231 and MCF-7 cells using lipofectamine 2,000 (Invitrogen) and X-tremeGENE siRNA transfection reagent (Roche Diagnostics, Mannheim, Germany) respectively. In brief, cells were plated in 6-well plates at a density of 3 × 10^5^ cells per well so they will be 90–95% confluent at the time of transfection. Lipofectamine 2,000 reagent or X-tremeGENE siRNA transfection reagent and plasmid were diluted in Opti-MEM^®^ Medium and mixed at 4:1 ratio incubating for 30 min at room temperature. Then, plasmid DNA-lipid complex was added to the cell culture medium. All transfected cells were verified by western blot.

### Coimmunoprecipitation Analysis

The Capturem ™ IP and CO-IP Kit (TaKaRa, Kyoto, Japan) was utilized to protein extraction, protein quantification, and protein incubation according to the manufacturer protocol. In brief, 2,000 μg protein (total 550 μl) were extracted from 1.5 × 10^7^ cells. 50 μl protein supernatants after boiled with 5× Laemmli SDS-sample buffer for 5 min were taken out as input group. The remaining 500 μl supernatant was re-incubated with ubiquitinated primary antibody of β-catenin (no. 66379-1-Ig) for 2 h at room temperature after subjected to shake with coupled antibody (no. 10201-2-AP) overnight at 4°C. The protein bands were visualized using the enhanced chemiluminescence detection reagents and quantified digitized by ImageJ software.

### Establishment of Animal Xenografts

To elucidate the chemosensitizing effects of ADQ *in vivo*, 4-week-old female BALB/C-nude mice were brought from Medical Experiment Center of Guangdong Province (Guangzhou, China) and all procedures were authorized by the Animal Care and Use Committee of Guangzhou University of Chinese Medicine. All the mice were housed in the SPF-level animal center at an ambient temperature between 21 and 25°C and a humidity between 50 and 60% with a 12 h light/dark cycle while food and water were offered sufficiently. 3 × 10^6^ MDA-MB-231-Luc cells were suspended in 100 μl of Matrigel matrix glue (Corning, Cambridge, MA, United States) and subcutaneously inoculated into the mammary fat pads of mice to establish xenograft models. When the tumor approximately grew to 5 × 5 mm, the mice were randomized into control group, ADQ formula group (100 mg/kg), taxol group (10 mg/kg) and taxol combined with ADQ formula group (*n* = 8). Taxol diluted with saline was intraperitoneally injected at 10 mg/kg every three days in taxol group or combination groups and ADQ group was given gavage at 100 mg/kg/day. Control group mice were given equal volume of saline by intragastric perfusion. The body weight and tumor sizes were monitored every 3 days throughout the whole experimental period. Subsequently, the tumor volume was estimated with formula [*V* = (length × width)^2^/2]. 150 mg/kg d-Luciferin (PerkinElmer, Boston, United States) was injected intraperitoneally into mice for *vivo* imaging observation with the IVIS Lumina XR imaging system (PerkinElmer). When the sizes of tumors grew to 15 × 15 mm, mice were sacrificed. The collected tumor tissues were fixed in 4% PFA for 12 h and then dehydrated and embedded in paraffin. Then processed samples were sectioned (4 μm) and applied for hematoxylin-eosin (HE) staining and immunohistochemistry (IHC) assay. To establish zebrafish xenotransplantation model, cells labeled with 1,1-Dioctadecyl-3,3,3,3-tetra-methylindocarbocyanine perchlorate (DiI, Sigma-Aldrich) and resuspended in serum-free DMEM medium (200 cells/20 nl), were microinjected into the AB strain zebra?sh, which were obtained from China Zebra?sh Resource Center (Wuhan, China). After 48 h, the zebrafishes treated above were then further incubated in 48-well plates at one fish per well with or without ADQ formula or taxol. Proliferation inhibitory effects of ADQ in zebrafish was inspected through fluorescence microscopy (Nikon Eclipse C1, Tokyo, Japan) and ImageJ software was applied for quantitative analysis.

### Hematoxylin-Eosin Staining

To better evaluate ADQ’s anti-cancer effects, H&E staining was performed with commercial Hematoxylin and Eosin Staining Kit (Beyotime) following the manufacturer’s instruction. Paraffin-embedded tumor sections, with 4 μm thick, were fixed onto poly-l-lysine-coated slides dewaxed twice with xylene for 10 min each, followed by gradual rehydration in 100–70% ethanol and then immersed in distilled water. Immediately after, 10% hematoxylin was performed to stain nuclear for 5 min followed by counterstaining cytoplasm with eosin for 2 min. After dehydration, hyalinization, sealed with neutral gum, the sections were baked, and pictures were taken and analyzed.

### Immunohistochemistry Analysis

For immunohistochemistry analysis, the slides of tumor tissues were deparaffinized twice with xylene for 10 min and rehydrated with 100–75% ethanol for 10 min. After washing with PBS three times, the slices were boiled in 10 mm sodium citrate buffer solution (pH 6.0, Solarbio, Beijing, China) for 8 min to perform antigen repair. To eliminate endogenous peroxidase activity, sections were permeabilized with 3% hydrogen peroxide dissolved in methanol at room temperature in the dark and then blocked by 10% goat serum to reduce nonspecific binding. The samples were then washed with PBS three times and incubated with a 1:100 diluted primary antibodies including β-catenin, GRP78, LC3 and ABCG2 in a humid chamber at 4°C overnight, followed by incubation with a 1:200 dilution of biotinylated secondary antibodies. Immediately thereafter, 3,3-diaminobenzidine substrate (DAB, ZSGB-BIO, Bejing, China) was applied for color development and a counterstain with Mayer’s hematoxylin was performed. Digital images of stained sections were taken with a BX46 Olympus microscope (Olympus, Center Valley, PA, United States).

### Statistical Analysis

All statistical analyses were performed with SPSS software (version 19.0, Abbott Laboratories, Chicago, United States) and plotted by GraphPad Prism 7.0. The data were represented as the mean ± standard deviation. Independent-Samples *t*-test was performed for the two groups of data in accordance with independence, normality and homogeneity of variance, and the One-Way ANOVA analysis was performed for comparison among multiple groups. Dunett T3 test was performed for samples with normal distribution but uneven variance. Rank sum test was used for the sample data that did not conform to the normal distribution. For all tests, *p* < 0.05 was considered to indicate a statistically significant difference.

## Results

### ADQ Exerts Anti-Cancer and Chemosensitivity Effects on Breast Cancer Cells

**FIGURE 1:**
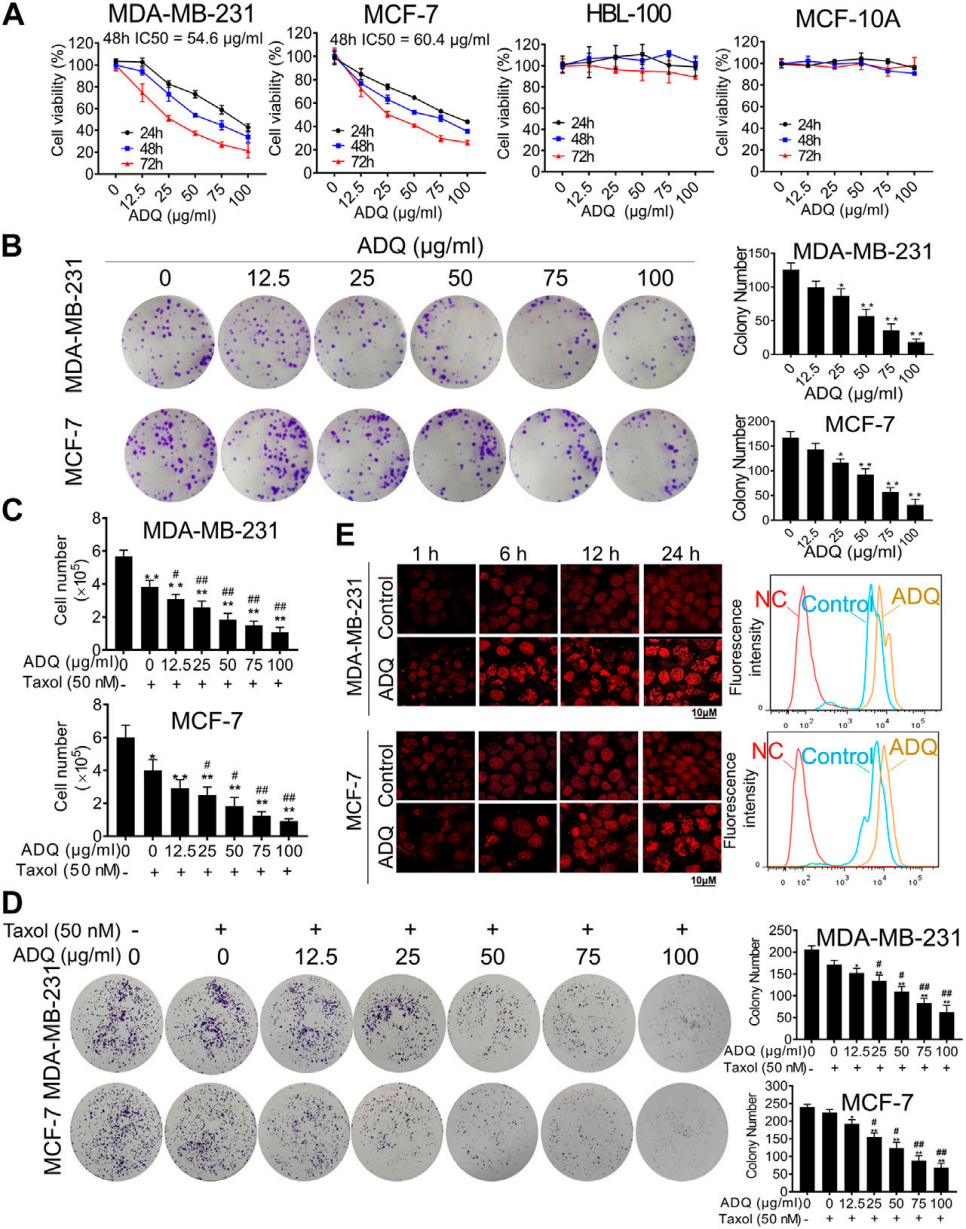
Ai Du Qing formula (ADQ) exerts anti-cancer and chemosensitivity effects on breast cancer cells. **(A)** CCK8 assay demonstrated that ADQ (0–100 μg/ml) exerted an inhibitory effect on breast cancer cells MDA-MB-231 and MCF-7, while posing little cytotoxicity on non-malignant mammary epithelial cell lines HBL-100 and MCF-10A. **(B)** ADQ exerted an obvious inhibition on the colony formation abilities of breast cancer cell lines MDA-MB-231 and MCF-7 at different concentrations (0–100 μg/ml). **(C)** Cell counting assay showed a synergistic effect of ADQ (0–100 μg/ml) with 50 nM taxol in MDA-MB-231 and MCF-7 cells. **(D)** Colony formation assay demonstrated synergistic effects of ADQ with taxol to suppress the colony size and number of MDA-MB-231 and MCF-7 cells. **(E)** Drug efflux assay demonstrated that ADQ (50 µg/ml) could increase the intake of epirubicin (10 µg/ml) in MDA-MB-231 and MCF-7 cells. All values represent the means ± SD (*n* =3, *p < 0.05, **p < 0.01 vs. Control group; ^#^ *p* < 0.05, ^##^ *p* < 0.01 vs. Taxol group).

The effects of ADQ on cell growth were validated using two human breast cancer cell lines, namely, MDA-MB-231, and MCF-7, as well as two human normal breast epithelial cell lines, namely, HBL-100, and MCF-10A. CCK-8 results revealed that ADQ suppressed the proliferation of both breast cancer cells in a dose- and time-dependent manner, while exerting obscure cytotoxic effects on HBL-100 and MCF-10A cells (Figure 1A). The IC_50_ values of ADQ in MDA-MB-231 were 83.844 μg/ml at 24 h, 54.604 μg/ml at 48 h, and 30.792 μg/ml at 72 h. However, its IC_50_ values for MCF-7 were 89.295 μg/ml at 24 h, 60.386 μg/ml at 48 h and 30.008 μg/ml at 72 h. In the colony formation assay, MDA-MB-231 and MCF-7 cells were treated with ADQ at concentrations ranging from 0 μg/ml to 100 μg/ml for 24 h and then cultured for an additional two weeks. As expected, the number and sizes of cell clones decreased with increasing ADQ concentration (Figure 1B). To further determine whether ADQ acts synergistically with traditional chemotherapeutics, we administered ADQ alone or together with the chemotherapeutic drug taxol at its IC_50_ value (50 nm) based on our pilot study (Zheng et al., 2019). Both cell counting and colony formation assays demonstrated that either ADQ or taxol alone could effectively limit the proliferation of MDA-MB-231 and MCF-7 cells, while its combination group resulted in a highly significant reduction compared to that using each separately (Figures 1C,D). In addition, the drug efflux assay indicated that epirubicin intake rapidly decreased in both ADQ-treated breast cancer cells, as indicated by attenuated fluorescence intensities (Figure 1E). Taken together, these results indicate that ADQ at non-cytotoxic doses effectively suppresses breast cancer cells, as well as enhances the chemosensitivity of breast cancer cells when administered in combination with taxol.

### ADQ Attenuates the Proportion, Self-Renewal, and Differentiation of Breast CSCs

**FIGURE 2:**
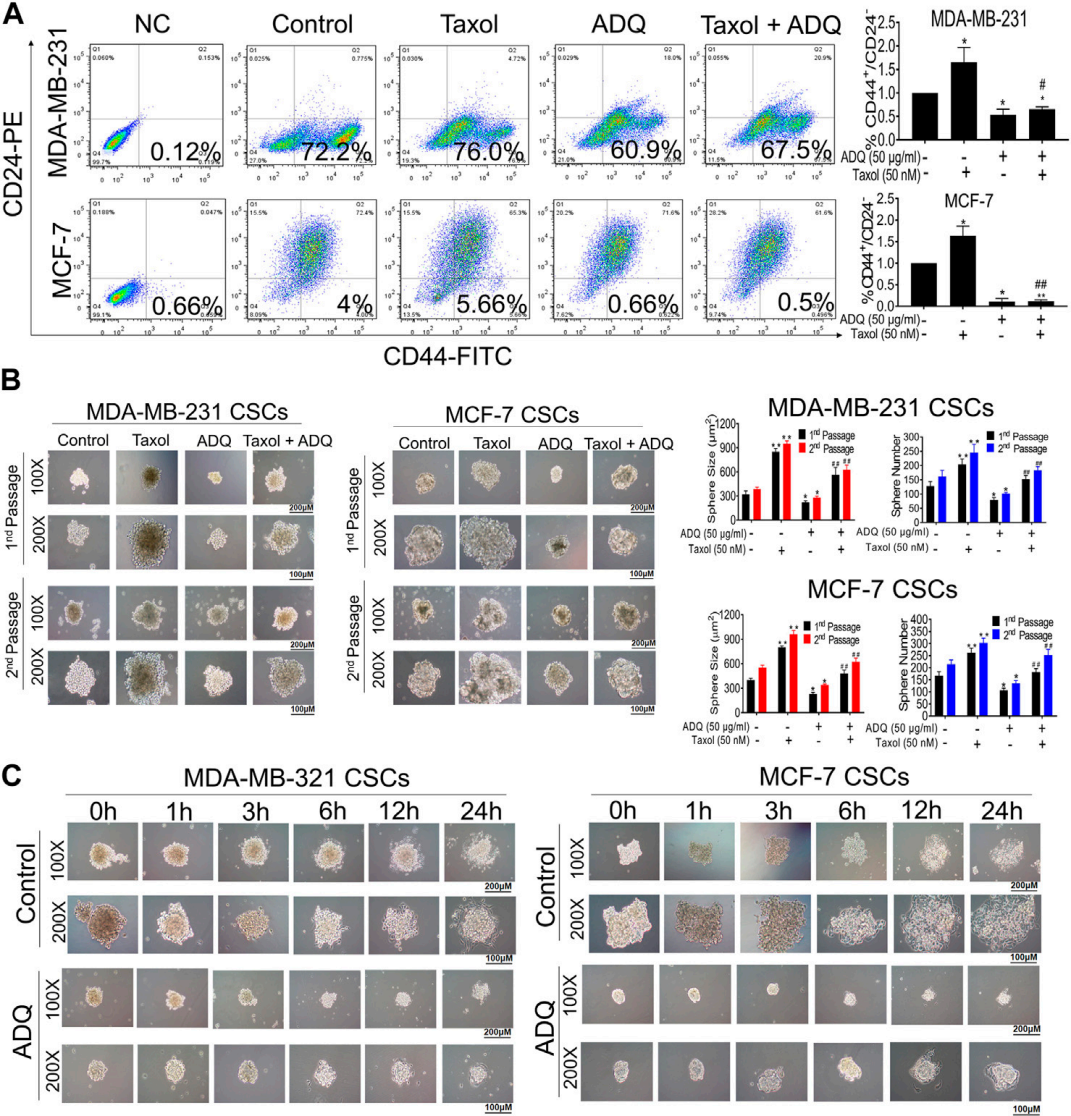
ADQ attenuates the proliferation, self-renewal and differentiation of breast CSCs. **(A)** ADQ administration for 48 h could remarkably reduce the proportions of CD44^+^CD24^−/low^ subsets in both the MDA-MB-231 cells and MCF-7 cells. **(B)** 50 μg/ml ADQ with or without 50 nM taxol markedly limited the numbers and sizes of the primary and secondary mammospheres. **(C)** ADQ treatment dramatically attenuated the differentiation ability of breast CSCs. All values represent the means ± SD (*n* = 3, *p < 0.05, **p < 0.01 vs. Control group; ^#^ *p* < 0.05, ^##^ *p* < 0.01 vs. Taxol group).

Breast cancer chemoresistance has been mainly associated with the presence of breast CSCs, which are characterized by unlimited self-renewal capacity and multidirectional differentiation potential (Bai et al., 2018). We first conducted flow cytometry to assess changes in the proportion of breast CSCs stained with CD44^+^/CD24^−/low^ after or before ADQ. We found that ADQ synergistically acts with taxol, resulting in a decrease in the CSC phenotype, which is characterized by CD44^+^/CD24^−/low^ in both MDA-MB-231 and MCF-7 cells. For example, in MDA-MB-231 cells, ADQ not only reduced CSC-like proportions from 72.2 to 60.9%, but also acted synergistically with taxol to suppress CD44^+^/CD24^−/low^ ratio (Figure 2A). Because breast CSCs always exist in the form of spheres, i.e., non-adherent spherical clusters of cells, we then examined the influence of ADQ on mammosphere formation in breast CSCs. As shown in Figure 2B, taxol administration increased the number and size of primary passage mammospheres in CSCs derived from MDA-MB-231 and MCF-7 cells. In contrast, ADQ administration not only remarkably suppressed the number and size of mammospheres but also weakened the taxol-promoting effects on breast CSCs spheres. Similar findings were also observed in the secondary-passage mammospheres, further validating the chemosensitizing effects of ADQ on breast CSCs. Given that multidifferentiation is another key feature of CSCs, we next evaluated the potential role of ADQ in the differentiation potential of MDA-MB-231 and MCF-7 CSCs. Prolongation of ADQ administration blocked CSC sphere differentiation, which implies that ADQ could also limit CSC differentiation potential (Figure 2C). Overall, ADQ could potently suppress the characteristics of CSCs, especially limiting its proliferation, self-renewal ability, as well as differentiation potential, which in turn results in cancer resistance.

### ADQ Abrogates Autophagy in Breast CSCs

**FIGURE 3:**
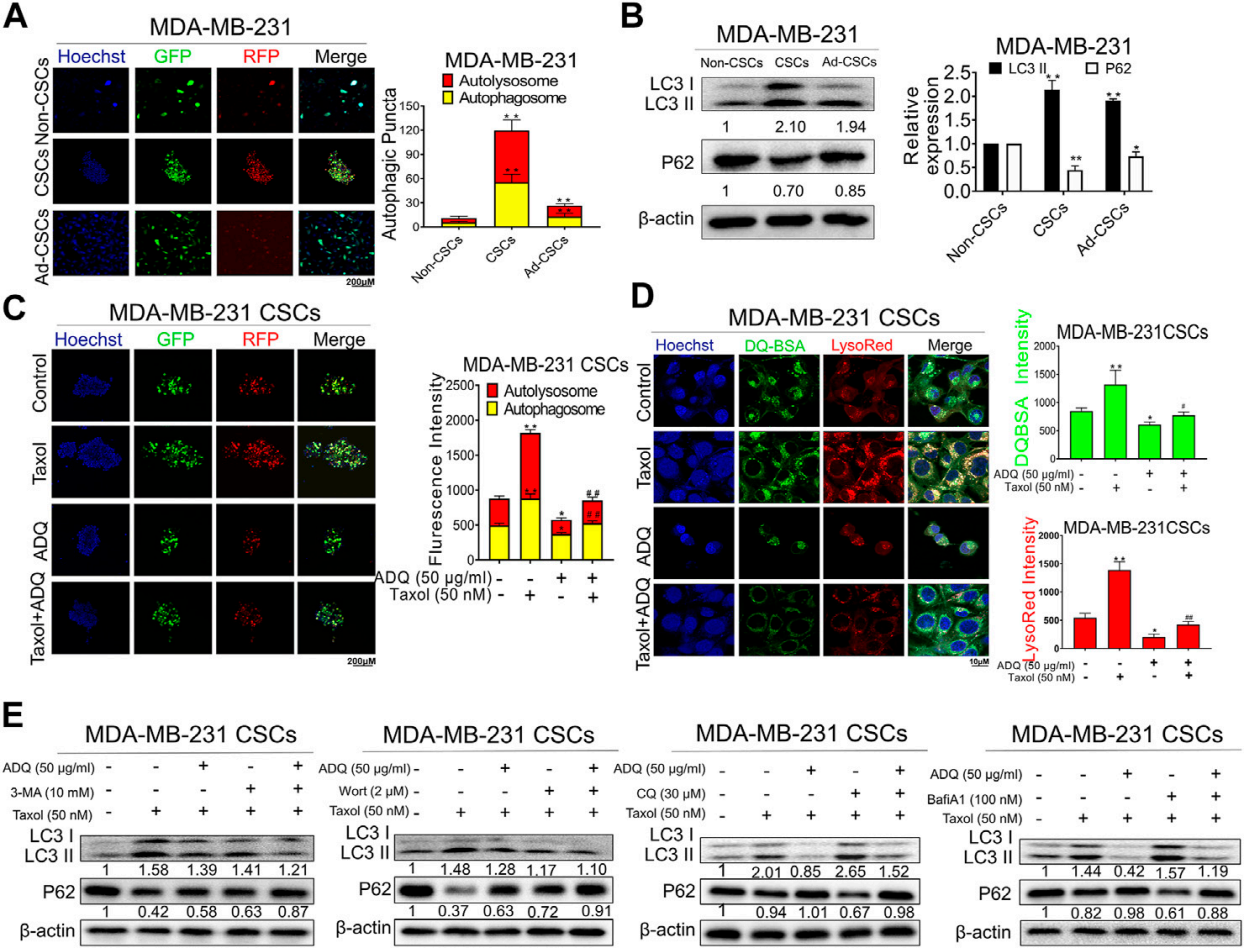
ADQ abrogates autophagy activity in breast CSCs. **(A)** Representative images of autophagic flux in breast CSC spheres, adherent breast cancer cells as well as differentiated breast CSCs. **(B)** The LC3 II and P62 expressions were analyzed by western blotting. All values represent the mean ± SD (*n* = 3, *p < 0.05, **p < 0.01 vs. Non-CSCs group). **(C)** Representative confocal images of autophagic flux in MDA-MB-231 CSC spheres transfected with the AVV-mRFP-GFP-LC3 reporter after the indicated treatment. All values represent the means ± SD (*n* = 3, *p < 0.05, **p < 0.01 vs. Control group; ^#^ *p* < 0.05, ^##^ *p* < 0.01 vs. Taxol group). **(D)** The fluorescence photographs of MDA-MB-231 CSC spheres were marked by DQ-BSA (green) or LysoRed (red) after the indicated treatment. All values represent the means ± SD (*n* = 3, *p < 0.05, **p < 0.01 vs. Control group; ^#^ *p* < 0.05, ^##^ *p* < 0.01 vs. Taxol group). **(E)** Representative bands of LC3 II and P62 in MDA-MB-231 CSCs with or without early autophagy inhibitor (3-mA, wortmannin) and late autophagy inhibitor (CQ, bafilomycin A1) after the indicated treatment.

CSCs are the source of chemotherapy resistance with relapse potential after traditional anti-cancer treatment (Bleau et al., 2009). Accumulating evidence has demonstrated that autophagy plays a crucial role in CSC activities (Han et al., 2018; Nazio et al., 2019). Thus, we continued to investigate the underlying role of autophagy in anti-CSCs and the chemosensitizing effects of ADQ. We first investigated the effects of ADQ on breast CSC autophagy using an AVV-mRFP-GFP-LC3-puro reporter, by which autolysosomes were indicated by yellow spots that overlapped with mRFP and GFP fluorescence, while autophagosomes were marked in free red puncta that indicate fusion of lysosomes and autophagosomes. Compared with either adherent breast cancer cells or differentiated breast CSCs, breast CSC spheres showed the most abundant yellow fluorescence, suggesting strongest autophagic activity in MDA-MB-231 (Figure 3A). This finding coincided with the results of western blotting analysis. The conversion of LC3 I to LC3 II in cells is considered a hallmark of autophagy formation, and P62 as an autophagy substrate is degraded as part of the autophagy process, and the reduction of P62 usually indicates the activation of autophagy (Wang et al., 2016). Our study revealed that breast CSC spheres presented higher LC3 II accumulation as well as lower P62 levels than adherent and differentiated cells (Figure 3B). Next, we investigated the combinatorial effects of ADQ and taxol on autophagy in breast CSCs using mRFP-GFP-LC3. As shown in Figure 3C, taxol administration led to an increase in both yellow and red dots, while additional ADQ treatment attenuated the taxol-promoting effects in autophagy, i.e., reduced the number of autophagosomes and autolysosomes in breast CSCs. To verify the effect of ADQ on the lysosomal activity of breast cancer cell lines, MDA-MB-231 cells were incubated with DQ-BSA and LysoRed followed by monitoring the level changes of autophagosomes and lysosomes. The results in Figure 3D demonstrated that ADQ markedly attenuated both LysoRed and DQ-BSA, suggesting that lysosomal degradation was blocked by ADQ. To further examine the stage-specific function of autophagy mediating the suppressing activities of ADQ on breast CSCs, early-stage autophagy inhibitors (3-MA, wortmannin) and late-stage autophagy inhibitors (CQ, Bafilomycin A1) were added to cultured MDA-MB-231 CSCs with either ADQ or taxol. As shown in Figure 3E, western blotting showed that the inhibitory effects of ADQ on LC3 II accumulation were not significantly influenced by the addition of either 3-MA or wortmannin, which suggests that ADQ treatment and the aforementioned upstream autophagy inhibitors possibly exerted similar activities in mediating autophagy. This was further supported by the fact that ADQ could attenuate the promotion of late-stage autophagy inhibitors CQ and Bafilomycin A1 on LC3-II conversion. Collectively, our data indicate that ADQ improved the chemosensitivity of breast CSCs to taxol possibly by abrogating early-phase autophagy activity.

### Ai Du Qing Activates the Akt/GSK3β-Mediated Proteasome Degradation of β-catenin in Breast Cancer Stem Cells

**FIGURE 4:**
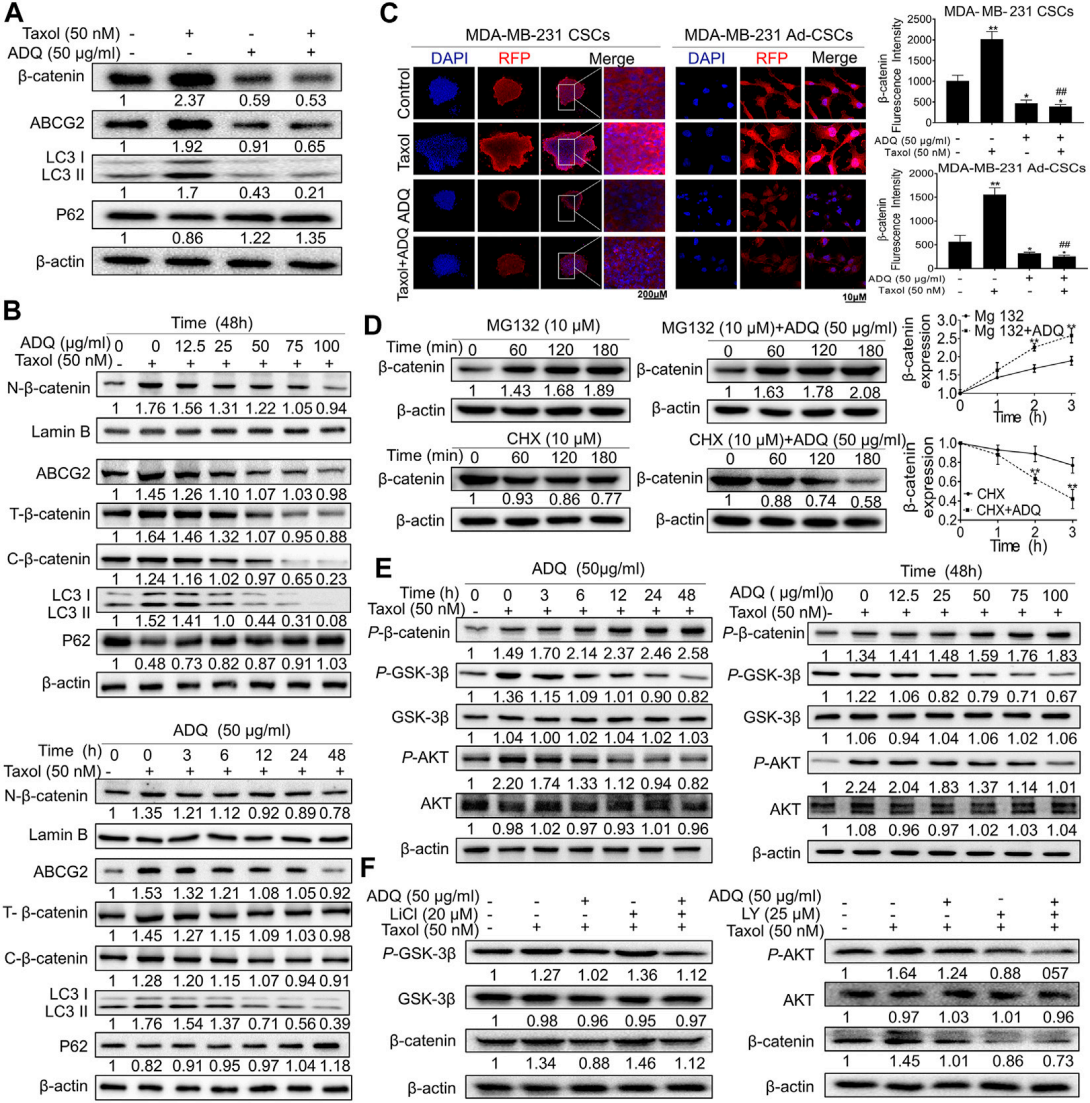
ADQ activates the Akt/GSK3β-mediated proteasome degradation of β-catenin in breast CSCs. **(A)** The expressions of β-catenin, ABCG2, P62 and LC3 in MDA-MB-231 CSCs were examined by western blotting after the indicated treatment. **(B)** The fractional or total expressions of β-catenin, ABCG2, P62 and LC3 in MDA-MB-231 CSCs were assayed by western blotting after the indicated treatment. **(C)** Representative immunofluorescent images of β-catenin in MDA-MB-231 CSCs spheres and its re-attached CSC cells. All values represent the means ± SD (*n* = 3, *p < 0.05, **p < 0.01 vs. Control group; ^#^ *p* < 0.05, ^##^ *p* < 0.01 vs. Taxol group). **(D)** The impacts of ADQ on the β-catenin proteasome degradation pathway were evaluated by western blotting. The upper graph shows that the action of ADQ on β-catenin degradation was blocked by the proteasome inhibitor MG132. The lower graph indicates the influence of ADQ on β-catenin degradation was accelerated by the protein synthesis inhibitor CHX. All values represent the means ± SD (*n* = 3, *p < 0.05, **p < 0.01 vs. Control group; ^#^ *p* < 0.05, ^##^ *p* < 0.01 vs. Taxol group). **(E)** Western blotting analysis demonstrated that ADQ administration notably affected the expressions of the phosphorylation of β-catenin, total/phosphorylation of GSK-3β as well as total/phosphorylation of AKT in a time- and dose-dependent manner. **(F)** Western blotting analysis showing the synergsitic effects of ADQ with either GSK-3β inhibitor LiCl or AKT inhibitor LY294002.

The molecular mechanisms addressing the autophagy-promoting effects of ADQ were next explored on breast CSCs. We previously demonstrated the abnormal activation of β-catenin and the subsequent upregulation of its downstream gene ABCG2 were tightly associated with drug-resistant properties of breast cancer (Wang N. et al., 2014). Hence, we next investigated the impact of ADQ on the expression and distribution of β-catenin in MDA-MB-231 CSCs. As shown in Figure 4A, western blotting demonstrated that the combination of ADQ and taxol resulted in the downregulation of β-catenin, ABCG2, and LC3 II and upregulation of P62 expression, indicating a positive correlation between β-catenin expression and autophagic makers. Furthermore, the inhibitory effects of ADQ on β-catenin, ABCG2, and LC3 II followed a time- and dose-dependent manner, and either nuclear or cytosolic β-catenin expression were simultaneously suppressed by ADQ (Figure 4B). Immunofluorescence assays were also performed to investigate the expressional pattern of β-catenin, and we observed that ADQ administration suppressed the accumulation of β-catenin, particularly nuclear translocation in either sphere form or re-attached form of MDA-MB-231 CSCs (Figure 4C). Given that proteasome degradation is the main regulatory mechanism that post-translationally regulates β-catenin expression, we next evaluated whether ADQ could activate proteasome degradation to downregulate β-catenin expression. As such, the proteasome inhibitor MG132 and the protein synthesis inhibitor cycloheximide (CHX) were added into the cultures of breast CSCs separately. MG132 treatment blocked ADQ-induced β-catenin degradation, which presented as increasing accumulation of β-catenin expression. In contrast, ADQ-mediated β-catenin degradation was aggravated following CHX administration, further implying that ADQ activates proteasome degradation of β-catenin (Figure 4D). GSK-3β is an upstream signal that promotes β-catenin degradation by inducing its phosphorylation form at the Ser33/Ser37/Thr41 site, and this process is also AKT-dependent. As an important substrate of AKT, GSK-3β activity is negatively regulated by AKT, which affects the stability of β-catenin by inhibiting the phosphorylation of glycogen synthase by GSK-3β (Sagredo et al., 2018). Therefore, the next step is to investigate the effects of ADQ on AKT/GSK-3β/β-catenin signaling. In our study, we found that the phosphorylation of β-catenin at these sites increased after ADQ treatment in a time- and dose-dependent manner, which is accompanied by the downregulated expression of *P*-GSK-3β and *P*-AKT (Figure 4E). This finding implied that ADQ promotes β-catenin degradation possibly by limiting the activity of AKT, thereby suppressing GSK-3β phosphorylation. To directly validate this theory, LY294002 (LY, AKT inhibitor) as well as lithium chloride (LiCl, GSK-3β inhibitor) were added to the culture system of ADQ-treated breast CSCs. As shown in Figure 4F, the inhibitory effect of ADQ on β-catenin was counteracted by LiC1 treatment, while LY294002 administration aggravated the degradation of β-catenin by ADQ, suggesting that ADQ activates the degradation pathway of β-catenin proteasome *via* Akt/GSK-3β signal transduction.

### GRP78 Decreases Breast Cancer Chemosensitivity Possibly Via Autophagy Induction of Breast Cancer Stem Cells

**FIGURE 5:**
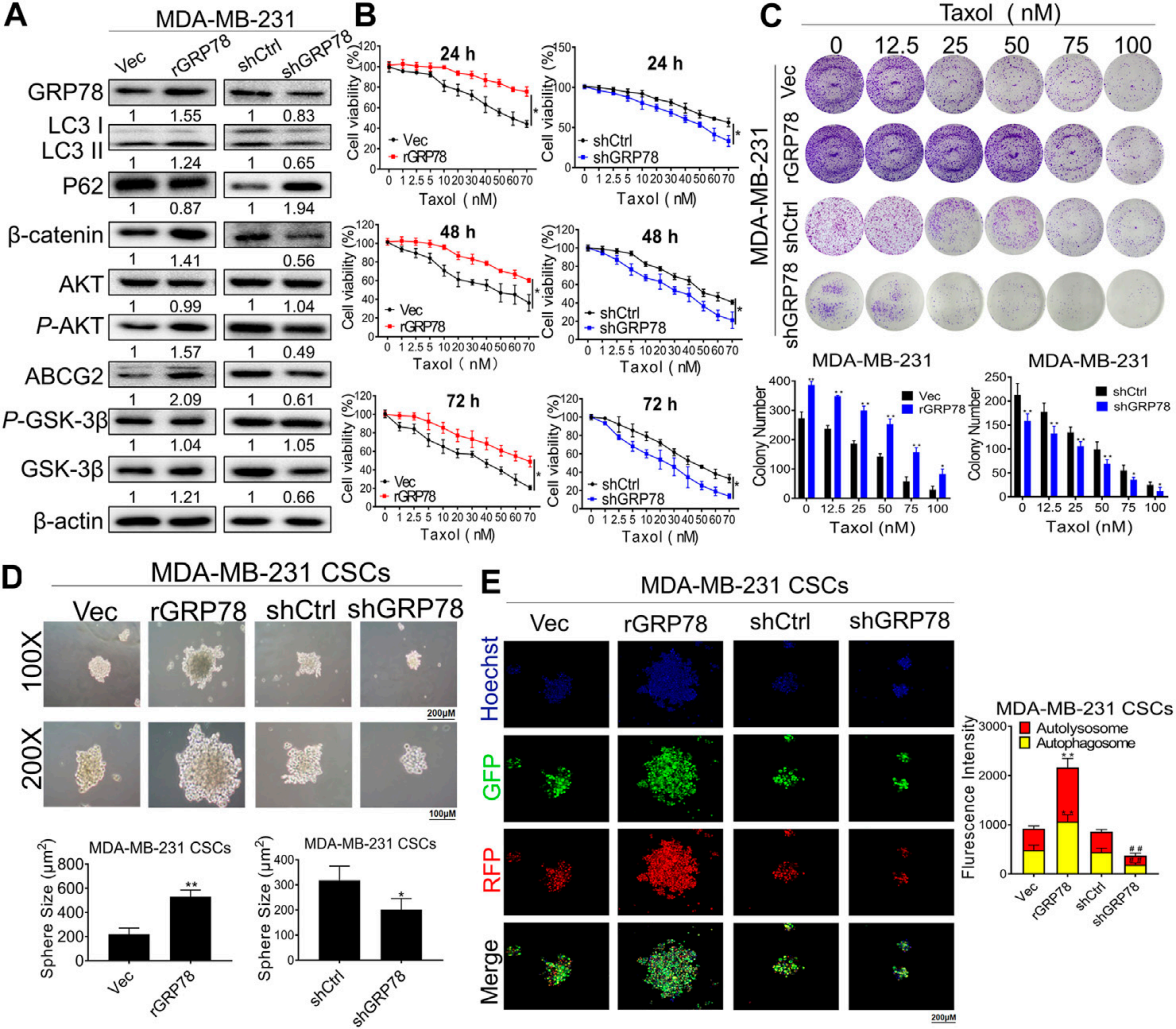
GRP78 decreases breast cancer chemosensitivity possibly via autophagy induction of breast CSCs. **(A)** Western blotting verified the expressions of GRP78, LC3, P62, β-catenin, ABCG2, GSK-3β, *P*-GSK-3β, AKT and *P*-AKT in MDA-MB-231 cells before or after the indicated transfection. **(B)** CCK8 assay detected the cell proliferation in GRP78^high^ and GRP78^low^ MDA-MB-231 cells with or without taxol administration. All values represent the means ± SD (*n* = 3, *p < 0.05, **p < 0.01 vs. Vec group; ^#^ *p* < 0.05, ^##^ *p* < 0.01 vs. shCtrl group). **(C)** Colony formation assay was performed to evaluate the long-term inhibitory effects of ADQ on GRP78^high^ and GRP78^low^ MDA-MB-231 cells. All values represent the means ± SD (*n* = 3, *p < 0.05, **p < 0.01 vs. Control group). **(D)** Sphere-forming assay in MDA-MB-231 CSCs before or after the indicated transfection. All values represent the means ± SD (*n* = 3, *p < 0.05, **p < 0.01 vs. Control group). **(E)** Fluorescence photographs of autophagic flux transfected with an LC3-GFP-mRFP reporter in GRP78^high^ and GRP78^low^ MDA-MB-231 cells. All values represent the means ± SD (*n* = 3, *p < 0.05, **p < 0.01 vs. Vec group; ^#^ *p* < 0.05, ^##^ *p* < 0.01 vs. shCtrl group).

Our pilot study identified GRP78 as a direct molecular target for breast CSC chemoresistance by stabilizing β-catenin (Wang N. et al., 2014). Moreover, network pharmacological analysis revealed that GRP78 is also one of hub targets of ADQ in the prevention and treatment of breast cancer. To investigate whether GRP78 is essential in mediating autophagy and drug resistance in breast CSCs, stable GRP78^high^ and GRP78^low^ MDA-MB-231 cells were constructed with lentivirus vector transfection for the following experiments. GRP78^high^ MDA-MB-231 cells exhibited enhanced LC3 II expression and decreased P62 levels, suggesting that GRP78 expression might be responsible for autophagic activation. Meanwhile, GRP78 overexpression triggered β-catenin/ABCG2 signaling possibly by upregulating the phosphorylation of AKT and GSK-3β, suggesting that the stabilization of β-catenin might be GRP78-depedent. Consistently, GRP78 silencing led to controversial results. As expected, GRP78 silencing not only led to a reduction in GRP78 expression, but also abolished autophagy-associated and β-catenin/ABCG2 signaling (Figure 5A). Therefore, we assumed that GRP78 might regulate autophagy *via* the β-catenin/ABCG2 signaling axis, consequently exerting chemoresistance to breast CSCs. To validate our assumption, the results of CCK-8 and clonogenic assays demonstrated that GRP78 overexpression improved the survival of taxol-treated breast cancer cells in a time- and dose-dependent manner, while GRP78 silencing apparently inhibited cell proliferation of taxol-treated breast cancer cells compared to the control group (Figures 5B,C). Furthermore, GRP78 promoted an increase in the number and size of breast CSC spheres (Figure 5D), as well as facilitated the upregulation of autophagic activity of breast CSCs (Figure 5E). In particular, red fluorescence puncta representing lysosomes and green fluorescence puncta representing autophagosomes were both enhanced in GRP78-overexpressing cells. In contrast, GRP78 silencing led to limited self-renewal capabilities and attenuated autophagic activities in MDA-MB-231 CSCs. Taken together, our findings demonstrate that GRP78 overexpression promotes self-renewal and proliferation, as well as decreases the chemosensitivity of breast CSCs possibly *via* autophagy induction.

### GRP78 Suppression by Ai Du Qing Formula Leads to β-Catenin Destabilization and Autophagy Inhibition in Breast Cancer Stem Cells

**FIGURE 6:**
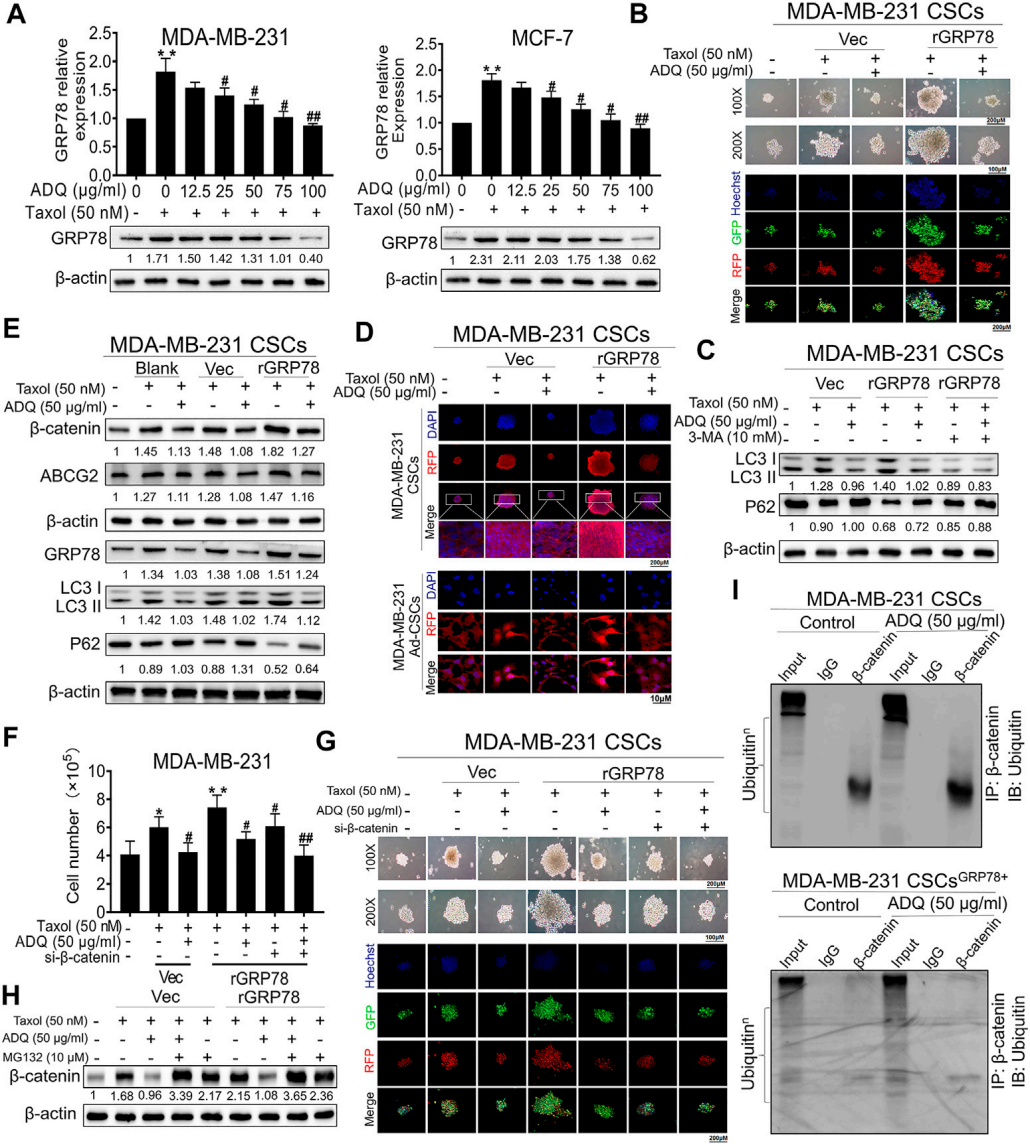
GRP78 suppression by ADQ leads to a β-catenin destabilization and an autophagy inhibition in breast CSCs. **(A)** Western blotting analysis showed that ADQ treatment for 48 h could inhibit GRP78 protein expression levels in MDA-MB-231 cells and MCF-7 cells in the presence of taxol. All values represent the means ± SD (*n* = 3, *p < 0.05, **p < 0.01 vs. Control group; ^#^ *p* < 0.05, ^##^ *p* < 0.01 vs. Taxol group). **(B)** Mammospheres assay and immunofluorescence analysis suggested that ADQ administration for 48 h could attenuate self-renewal and autophagic activities in GRP78^high^ MDA-MB-231 CSCs. **(C)** Western blotting analysis demonstrated that the autophagy activity in GRP78-overexpressed breast CSCs was significantly limited by the combination of 3-MA and ADQ. **(D)** Immunofluorescence experiments showed that ADQ not only limited the transfer of β-catenin from the cytoplasm to the nucleus, but also decreased the expression of β-catenin in GRP78^high^ MDA-MB-231 CSCs. **(E)** ADQ treatment for 48 h could attenuate β-catenin and ABCG2 expressions in GRP78^high^ MDA-MB-231 CSCs. **(F)–(G)** Cell number and sphere formation assay using GRP78^high^ MDA-MB-231 CSCs transfected with or without siβ-catenin after the indicated treatment. All values represent the means ± SD (*n* = 3, *p < 0.05, **p < 0.01 vs. Control group; ^#^ *p* < 0.05, ^##^ *p* < 0.01 vs. Taxol group). **(H)** Western blotting assay showing the effects of ADQ on the β-catenin proteasome degradation pathway in GRP78^high^ MDA-MB-231 CSCs. **(I)** Co-immunoprecipitation and ubiquitination array revealed that GRP78 overexpression reduced poly-ubiquitination accumulation of β-catenin in breast CSCs, while ADQ administration notably promoted the ubiquitination of β-catenin in breast CSCs.

We continued to examine whether β-catenin destabilization and autophagy inhibition by ADQ is GRP78-dependent in breast CSCs. Compared to taxol-treated group, GRP78 expression gradually decreased in the presence of ADQ in a concentration-dependent manner in MDA-MB-231 and MCF-7 cells (Figure 6A). To evaluate the role of GRP78 in the anti-autophagic and anti-CSCs capabilities of ADQ, we observed that GRP78 overexpression accelerated the self-renewal of breast CSCs and increased the number of yellow fluorescence puncta that represented the production of autophagosomes, which was attenuated by ADQ (Figure 6B). We continued to examine whether autophagic inhibition influenced the GRP78-decreasing effects on breast CSCs. The autophagy activity in GRP78-overexpressed breast CSCs was significantly limited by the combination of 3-MA and ADQ (Figure 6C). To investigate the regulation of GRP78 by ADQ on β-catenin distribution and expression, we performed immunofluorescence analysis of β-catenin in GRP78-overexpressed breast CSCs and found that GRP78 promotes β-catenin transport from the cytoplasm to the nucleus, whereas this phenomenon was reversed by ADQ treatment (Figure 6D). Meanwhile, western blotting analysis showed that ADQ decreased the taxol-induced expression of β-catenin and ABCG2, while additional GRP78 expression abolished the downregulating effects of ADQ (Figure 6E). To directly determine the critical role of β-catenin on ADQ action, we then downregulated β-catenin levels by transfecting its siRNA to the GRP78-overexpressing breast CSC culture system of MDA-MB-231. After β-catenin silencing, the proliferation, self-renewal and autophagy activities of GRP78-overexpressing breast CSCs were suppressed in the presence of ADQ, implying a positive regulatory relationship between GRP78 and β-catenin (Figures 6F,G). We next explored whether the proteasome degradation of β-catenin by ADQ was attributable to GRP78 activation. Compared with the Vec group, β-catenin accumulation was accelerated in GRP78-overexpressing breast CSCs when the proteasome degradation pathway was inhibited by MG132, indicating that proteasome degradation of β-catenin by ADQ might be largely attributable to GRP78 expression (Figure 6H). We further performed co-immunoprecipitation (CoIP) combined with ubiquitination array in either CSCs^Vec^ or CSCs^GRP78^ in the presence of ADQ. As shown in Figure 6I, ADQ notably enhanced the ubiquitination of β-catenin in both MDA-MB-231 CSCs and MDA-MB-231 CSCs^GRP78^. Decreased ubiquitination of β-catenin was observed in MDA-MB-231 CSCs^GRP78^ when compared to MDA-MB-231 CSCs group, further verifying that the ubiquitination pathway of β-catenin was suppressed by GRP78. Western blotting demonstrated that ADQ abolished the expression of GRP78 and ABCG2, as well as offset the induction effect of taxol on their expressions, indicating that GRP78 mediated ADQ-induced chemosensitivity (Supplementary Figure S1A). The cell counting assay showed that either ADQ or taxol alone effectively limits cell growth in MDA-MB-231 and MCF-7 cells, while its combination resulted in a highly significant reduction in comparison with each component (Supplementary Figure S1B). These data suggest that ADQ could reverse the chemical resistance induced by GRP78 at the protein translation level. To explore the molecular mechanism by which ADQ downregulated GRP78 expression, we further examined GRP78 mRNA levels in MDA-MB-231 and MCF-7 cells with ADQ treatment. We found that ADQ administration had little influence on GRP78 mRNA levels, suggesting that the suppression of GRP78 by ADQ might be posttranslational rather than transcriptional (Supplementary Figure S1C). Overall, ADQ influences the β-catenin/ABCG2 signaling axis *via* GRP78 to mediate autophagy, thereby increasing the sensitivity of breast CSCs to chemotherapeutic drugs.

### Ai Du Qing Enhances the Chemosensitivity of Taxol to Breast Cancer in Nude Mice and Zebrafish Breast Cancer Xenograft Models

**FIGURE 7:**
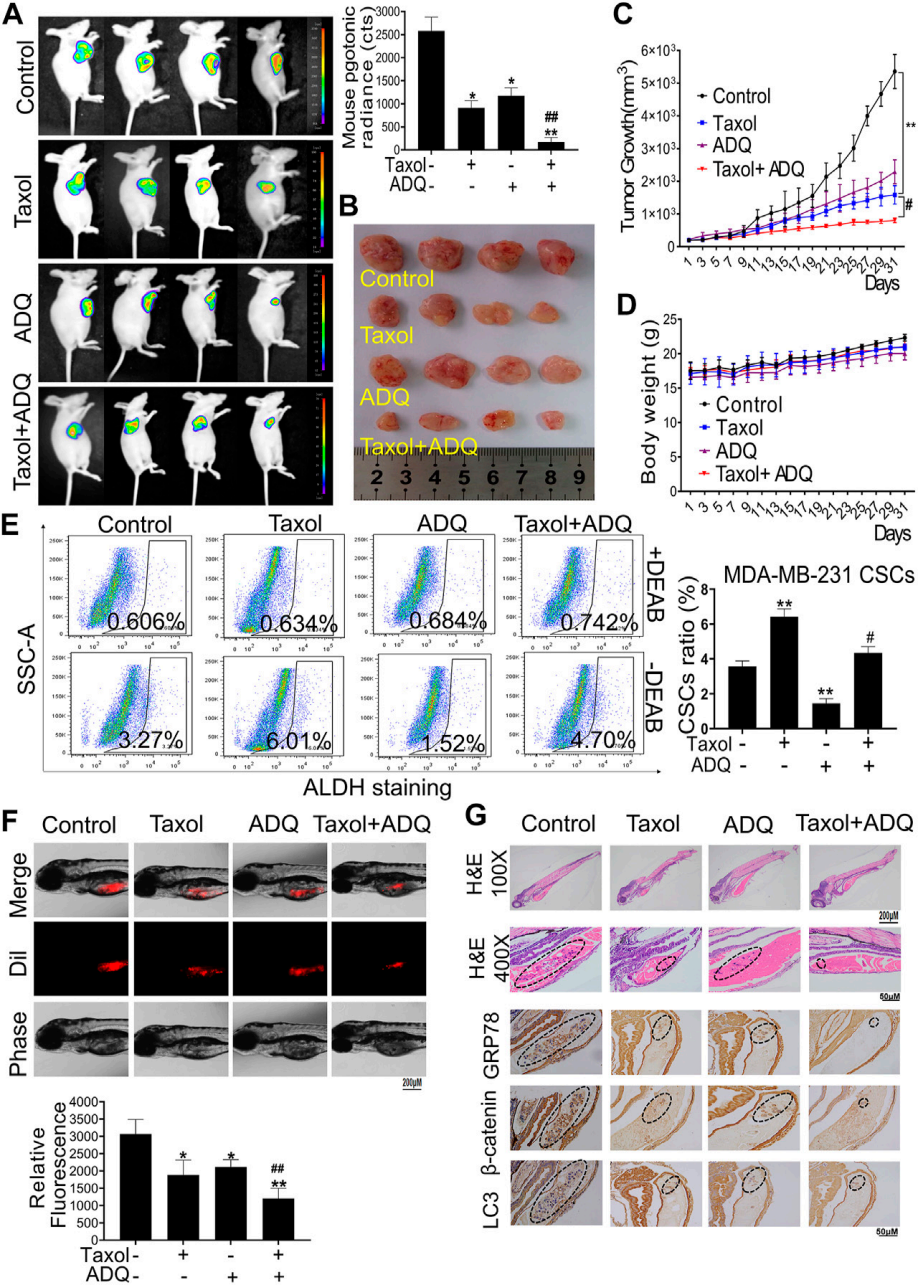
ADQ enhances the chemosensitivity of breast cancer in *ex vivo* and *in vivo* validation. **(A)** Representative bioluminescent images of MDA-MB-231-Luc xenograft mice model. Breast cancer xenografts were established by implanting luciferase-labeled MDA-MB-231-Luc cells into the mammary glands of BALB/C mice. Mice bearing MDA-MB-231-Luc xenografts received either saline or ADQ (100 mg/kg/day) by intragastric perfusion. All values represent the means ± SD (*n* = 4, *p < 0.05, **p < 0.01 vs. Control group; ^#^ *p* < 0.05, ^##^ *p* < 0.01 vs. Taxol group). **(B,C)** ADQ synergistically interacted with taxol to inhibit tumor growth in the MDA-MB-241-Luc xenograft model *in vivo*. All values represent the means ± SD (*n* = 4, *p < 0.05, **p < 0.01 vs. Control group; ^#^ *p* < 0.05, ^##^ *p* < 0.01 vs. Taxol group). **(D)** There were no significant differences in mouse body weights between the saline group and ADQ group, indicating no additional toxic and side effects of ADQ. *n* = 8. **(E)** ADQ administration obviously diminished the proportions of ALDH^+^ subsets induced by taxol. All values represent the means ± SD (*n* = 4, *p < 0.05, **p < 0.01 vs. Control group; ^#^ *p* < 0.05, ^##^ *p* < 0.01 vs. Taxol group). **(F)** ADQ (50 μg/ml) remarkably enhanced the inhibitory effects of Taxol (50 nM) on the zebrafish models bearing Dil-labled MDA-MB-231 cells. The red numbers represent the mean fluorescence intensities of Dil-stained MDA-MB-231 cells. All values represent the means ± SD (*n* = 4, *p < 0.05, **p < 0.01 vs. Control group; ^#^ *p* < 0.05, ^##^ *p* < 0.01 vs. Taxol group). **(G)** Representative images of H&E and representative IHC images of the expression levels of GRP78, β-catenin, and LC3 in zebrafish models.

To support the aforementioned *in vitro* findings, we next validated the anti-breast cancer activities and chemosensitization mechanism of ADQ *in vivo*. First, we established breast cancer xenografts by injecting luciferase-labeled MDA-MB-231-Luc cells for monitoring the anti-tumor and chemosensitizing effects of ADQ. ADQ was given once daily by gastric perfusion at 100 mg/kg/day, and taxol was diluted with saline and administered by intraperitoneal injection at 10 mg/kg every three days (Wang et al., 2018). As shown in *in-vivo* bioluminescent imaging, either taxol or ADQ alone could lead to suppression of tumor growth, while the combination of taxol and ADQ exerted the strongest inhibitory effect (Figures 7A–C). Compared with the control group, there was no significant weight loss or death of nude mice in each experimental group, indicating that ADQ had no obvious toxic side effects on nude mice (Figure 7D). We next explored the inhibitory effects of ADQ on CSC proportions *in vivo*. The activity of aldehyde dehydrogenase (ALDH) activity indicates the characteristics of CSCs, and the ALDH^+^ subpopulation has stronger stemness than the ALDH- subpopulation (Sun et al., 2020). As indicated in Figure 7E, the proportion of ALDH^+^ subpopulation in the synergistic group was apparently smaller than that in the control and taxol-treated groups, suggesting that ADQ could suppress the ratio of breast CSCs promoted by taxol administration *in vivo*. We also constructed a zebrafish xenograft model by microinjecting MDA-MB-231 CSCs stained with Dil marker to monitor the anti-tumor and chemosensitizing effects of ADQ. The results implied that the combination of taxol and ADQ showed the most obvious effects in suppressing the growth of tumors compared with either taxol or ADQ alone (Figure 7F). Furthermore, the results of immunohistochemical staining showed that the combination of ADQ and taxol could remarkably downregulate the expression of GRP78, β-catenin, and LC3 II, suggesting that ADQ might be developed as a novel chemosensitizing reagent with anti-autophagic activities (Figure 7G). Taken together, these results demonstrate that ADQ suppresses autophagy-promoting tumor growth by inhibiting the GRP78/β-catenin/ABCG2 axis *in vivo*, thereby increasing the chemosensitivity of breast CSCs to taxol.

## Discussion

Continuous in-depth studies on traditional Chinese and Western medicine have shown that TCM has great potential and unique advantages in combating breast cancer such as reversing tumor drug resistance, alleviating side effects, and exerting synergistic effects with traditional strategy (Zhang et al., 2020). Here, we demonstrate that a traditional TCM formula ADQ at safe dosages could remarkably chemosensitize breast CSCs by autophagic inhibition *in vivo* and *in vitro* (Wang et al., 2018). Our pilot study identified 132 candidate compounds in ADQ interacting with 22 core targets involved in chemoresistant breast cancer (*p* ≤ 0.05, FC ≥ 1.5) (Wang et al., 2018). Among these core compounds of ADQ, quercetin, 7-methoxy-2-methyl isoflavone, and *p*-coumaric acid were found be closely associated with the target GRP78 (Sharma et al., 2018; Cao et al., 2019; Su et al., 2019). Furthermore, our pilot HPLC analysis demonstrated that p-coumaric acid, curcumol, liquirtin, calycosin-7-glucoside, and glycyrrhizic acid are the major bioactive components in ADQ (Wang et al., 2018). The possible active compound p-coumaric acid was then obtained by taking the intersection of network pharmacology and HPLC analysis. Retrospectively, p-coumaric acid not only inhibited tumor cell proliferation but also attenuated CSCs, which were tightly associated with endoplasmic reticulum stress (ER) and autophagy. P-coumaric acid was reported to exert its anti-proliferative effect by downregulating GRP78 and activating unfolded protein response (UPR)-mediated apoptosis *in vitro* and *in vivo* (Sharma et al., 2018). P-coumaric acid treatment was also found to significantly suppress tumor growth in a xenograft model by downregulating stem cell markers and β-catenin as well as HIF-1α signaling (Min et al., 2015). Further mechanistic insights demonstrated that autophagy is a novel molecular mechanism that is involved in the crosstalk between classical actions of p-coumaric acid and exerted alternative therapeutic pathways for this compound (Abazari et al., 2021). Thus, we will continue to explore the anti-CSC as well as autophagic inhibition effects of p-coumaric acid on breast cancer in the future.

CSC activation requires a basic level of autophagy, and autophagy-promoting regulation could mediate the growth and pluripotency of CSCs (Sharif et al., 2017). Autophagy is activated upon cellular stress or nutrient deprivation to maintain cell survival *via* degradation of intracellular proteins, disrupting organelles, and energy storage, thus preventing cells from being damaged upon endoplasmic reticulum stress and oxidative stress (Liang et al., 2020). However, autophagy could induce autophagic apoptosis of CSCs in anti-tumor therapy, serving as an antagonistic role in protecting CSCs from resisting the toxicity of chemotherapy drugs in drug-resistant tumor cells (Huang et al., 2018; Nazio et al., 2019; Yang et al., 2020). In this study, we demonstrated that ADQ exerts an inhibitory effect on taxol-induced autophagy, subsequently chemosensitizing breast CSCs. Interestingly, we found that ADQ shared a similar regulatory mechanism with either 3-MA or wortmannin by suppressing early-phase autophagy, while exerting an additional effect with CQ and Bafilomycin A1 in mediating late-stage autophagy. This finding implicated that ADQ might be an early-phase autophagy inhibitor that suppresses breast CSC autophagy and chemoresistance. Currently, multiple anti-autophagy compounds have been identified, but only late-phase autophagic inhibitors chloroquine (CQ) and its derivative hydroxychloroquine (HCQ) have been approved for clinical application (Du et al., 2020). Nevertheless, their off-target efficacy and toxicity largely limits their clinic advancement. Because ADQ acts synergistically with CQ on autophagy activity, it is worthwhile to investigate whether ADQ could promote the efficacy of these two clinical anti-autophagy agents while suppressing their toxicity in our future investigation.

The glucose-regulated protein 78-kDa GRP78 is an endoplasmic reticulum stress protein located on chromosome 9q33 and contains eight exons and seven introns and consists of an N-terminal domain containing ATPase catalytic site and C-terminal substrate binding region (Bi et al., 2018). Overexpression of GRP78 induces resistance in tumors to various therapeutic drugs, including chemotherapeutic drugs, antihormonal drugs, DNA damaging agents, and anti-angiogenic factors, while the sensitivity of tumor cells to chemotherapy is significantly enhanced after GRP78 inhibition (Chen et al., 2018). Clinical studies have shown that patients overexpressing GRP78 had a high recurrence rate and shorter recurrence duration particularly when stage II and III breast cancer are treated with doxorubicin (Direito et al., 2019). More importantly, our pilot study identified that GRP78 is a novel target that is closely related to breast CSC resistance by regulating β-catenin/ABCG2 signaling, which highlights the significance of GRP78 in mediating CSCs resistance (Wang N. et al., 2014). GRP78 promotes self-renewal in CSCs, as well as upregulates the expression of CSC markers, including the ALDH1 and CD44, thereby contributing to cancer recurrence and resistance to chemotherapy (Hu et al., 2017). In our investigation, in comparison with either adherent breast cancer cells or differentiated breast CSCs, breast CSC spheres showed the strongest autophagic activity (Figures 3A,B), suggesting that autophagy mediates activities of breast CSCs, particularly in terms of resistance to chemotherapy. This finding is concordant to a series of studies on the role of autophagy in cancer progression. The activation of autophagy significantly promoted the percentage of glioma CSCs and their self-renewal ability, thus improving the chemotherapy resistance of glioma CSCs to temozolomide (Abbas et al., 2020). Autophagy was upregulated in some CSCs and played a crucial role in tumor growth and resist conventional treatments (Camuzard et al., 2020). These findings imply a possible association between GRP78 and breast CSCs, and thus we continued to investigate whether GRP78 would be essential in mediating autophagy and drug resistance in breast CSCs in this study. Here, we constructed the stable GRP78^high^ and GRP78^low^ MDA-MB-231 cells to investigate whether GRP78 is essential in mediating autophagy and drug resistance in breast CSCs. Western blotting and GFP-mRFP-LC3 immunofluorescence revealed that GRP78 overexpression leads to an increase in microtubule-associated light chain 3-II (LC3-II) conversion as well as enhanced autophagic flux in breast CSC spheres (Figures 5A,E). In addition, GRP78 silencing led to absolutely controversial results. Further western blotting analysis showed that ADQ decreased taxol-induced β-catenin and ABCG2 expression, while additional GRP78 expression abolished such downregulating effects of ADQ (Figure 6E). In terms of mechanism exploration, co-immunoprecipitation combined with ubiquitination assay verified that GRP78 stabilizes the ubiquitination of β-catenin, while ADQ notably accelerates the ubiquitination of β-catenin (Figure 6I). Studies on the association between CSCs and autophagy are limited, and thus the current study aimed to address this issue by investigating the role of GRP78 in mediating breast CSC autophagy *via* β-catenin/ABCG2 signaling, which subsequently leads to chemosensitivity in the presence of ADQ.

## Conclusion

In sum, these results demonstrate that ADQ suppresses breast cancer growth by inhibiting autophagy *via* the GRP78/β-catenin/ABCG2 axis, thereby increasing the chemosensitivity of breast CSCs to taxol. This study has elucidated the chemosensitizing molecular mechanism of ADQ in the treatment of breast cancer as well as highlights the importance of GRP78 in mediating β-catenin signaling and cancer drug resistance in CSCs.

## Data Availability

The original contributions presented in the study are included in the article/Supplementary Material, further inquiries can be directed to the corresponding authors.
