# Corrigendum: Autophagy Blockade by Ai Du Qing Formula Promotes Chemosensitivity of Breast Cancer Stem Cells Via GRP78/β-Catenin/ABCG2 Axis

**DOI:** 10.3389/fphar.2022.809565

**Published:** 2022-02-15

**Authors:** Mianmian Liao, Caiwei Wang, Bowen Yang, Danping Huang, Yifeng Zheng, Shengqi Wang, Xuan Wang, Juping Zhang, Chunbian Tang, Zheng Xu, Yu He, Ruolin Huang, Fengxue Zhang, Zhiyu Wang, Neng Wang

**Affiliations:** ^1^ The Research Center for Integrative Medicine, School of Basic Medical Sciences, Guangzhou University of Chinese Medicine, Guangzhou, China; ^2^ Department of Medical Biotechnology, School of Basic Medical Sciences, Guangzhou University of Chinese Medicine, Guangzhou, China; ^3^ Integrative Research Laboratory of Breast Cancer, The Second Clinical College, Guangzhou University of Chinese Medicine, Guangzhou, China; ^4^ Guangdong Provincial Key Laboratory of Clinical Research on Traditional Chinese Medicine Syndrome, Guangdong Provincial Academy of Chinese Medical Sciences, Guangdong Provincial Hospital of Chinese Medicine, Guangzhou, China; ^5^ Shenzhen Clinical Medical College, Guangzhou University of Chinese Medicine, Guangzhou, China; ^6^ Department of Hepatology, Shenzhen Traditional Chinese Medicine Hospital, The Fourth Clinical Medical College of Guangzhou University of Chinese Medicine, Shenzhen, China

**Keywords:** breast cancer chemosensitivity, cancer stem cells, autophagy, Ai Du Qing formula, GRP78/β-catenin/ABCG2 axis

In the original article, there were mistakes in Figures 1, 2, 5 as published. Figure 1E inadvertently contained duplicate images. In Figures 2B,C, certain spheres were unintentionally misplaced during picture combination. In Figure 5D, the ×200 sphere image of shCtrl was also unintentionally misplaced. The authors provided the journal with the original data files. The corrected figures, produced from the original data, appear below.

**FIGURE 1:**
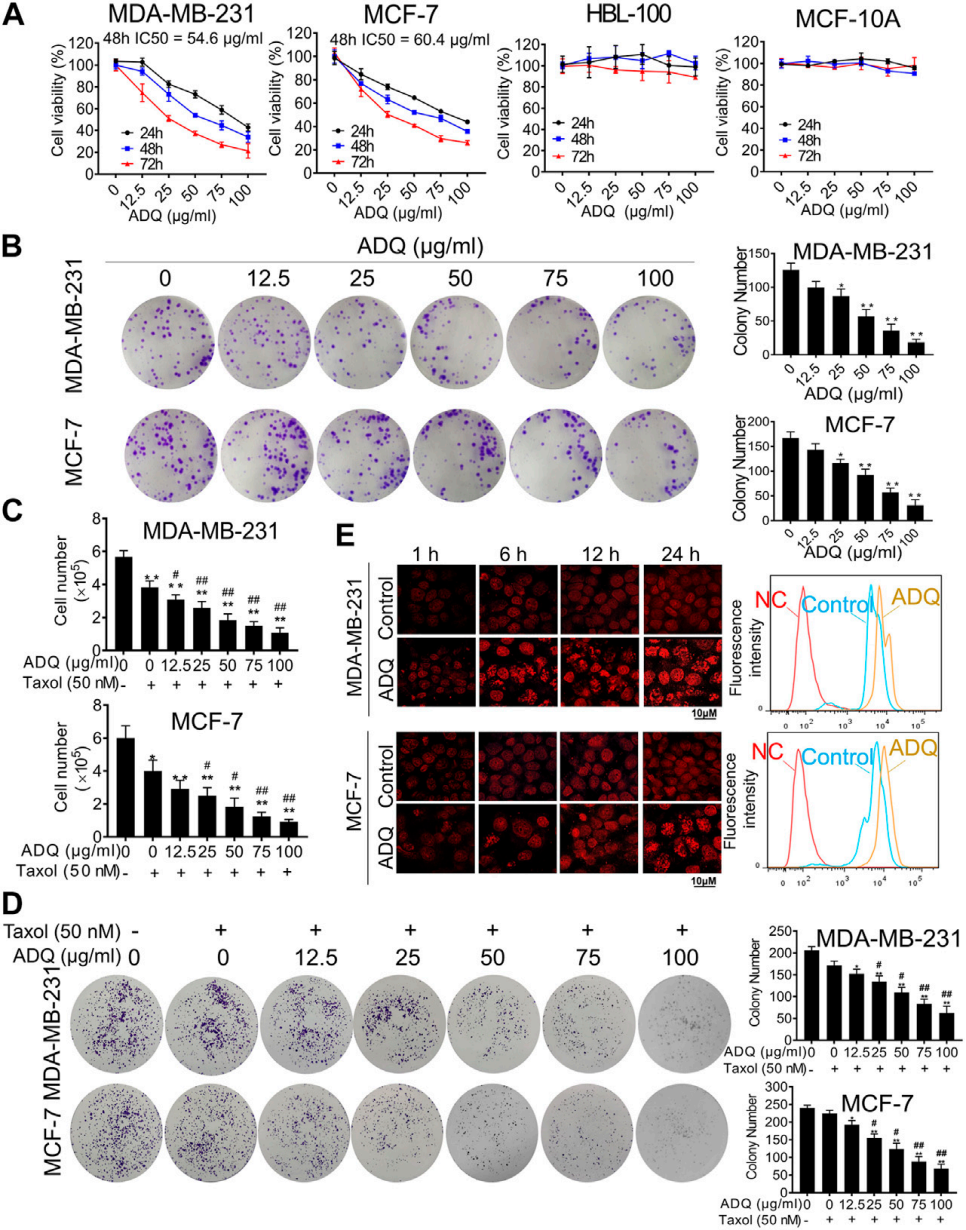
Ai Du Qing formula (ADQ) exerts anti-cancer and chemosensitivity effects on breast cancer cells. **(A)** CCK8 assay demonstrated that ADQ (0–100 μg/ml) exerted an inhibitory effect on breast cancer cells MDA-MB-231 and MCF-7, while posing little cytotoxicity on non-malignant mammary epithelial cell lines HBL-100 and MCF-10A. **(B)** ADQ exerted an obvious inhibition on the colony formation abilities of breast cancer cell lines MDA-MB-231 and MCF-7 at different concentrations (0–100 μg/ml). **(C)** Cell counting assay showed a synergistic effect of ADQ (0–100 μg/ml) with 50 nM taxol in MDA-MB-231 and MCF-7 cells. **(D)** Colony formation assay demonstrated synergistic effects of ADQ with taxol to suppress the colony size and number of MDA-MB-231 and MCF-7 cells. **(E)** Drug efflux assay demonstrated that ADQ (50 μg/ml) could increase the intake of epirubicin (10 μg/ml) in MDA-MB-231 and MCF-7 cells. All values represent the means ± SD (*n* = 3, *p < 0.05, **p < 0.01 vs. Control group; #*p* < 0.05, ##*p* < 0.01 vs. Taxol group).

**FIGURE 2:**
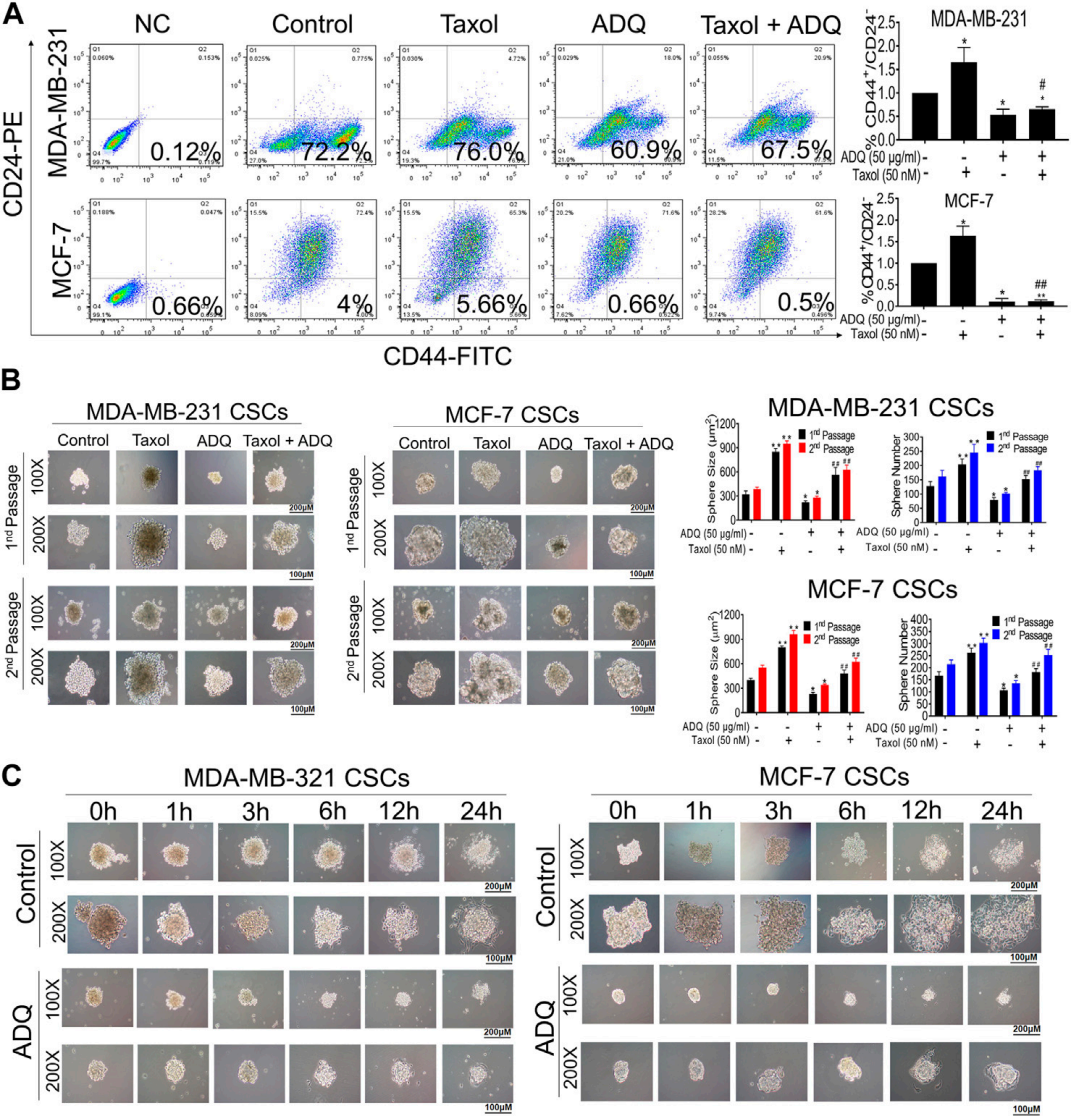
ADQ attenuates the proliferation, self-renewal and differentiation of breast CSCs. **(A)** ADQ administration for 48 h could remarkably reduce the proportions of CD44^+^CD24^−/low^ subsets in both the MDA-MB-231 cells and MCF-7 cells. **(B)** 50 μg/ml ADQ with or without 50 nM taxol markedly limited the numbers and sizes of the primary and secondary mammospheres. **(C)** ADQ treatment dramatically attenuated the differentiation ability of breast CSCs. All values represent the means ± SD (*n* = 3, *p < 0.05, **p < 0.01 vs. Control group; #*p* < 0.05, ##*p* < 0.01 vs. Taxol group).

**FIGURE 5:**
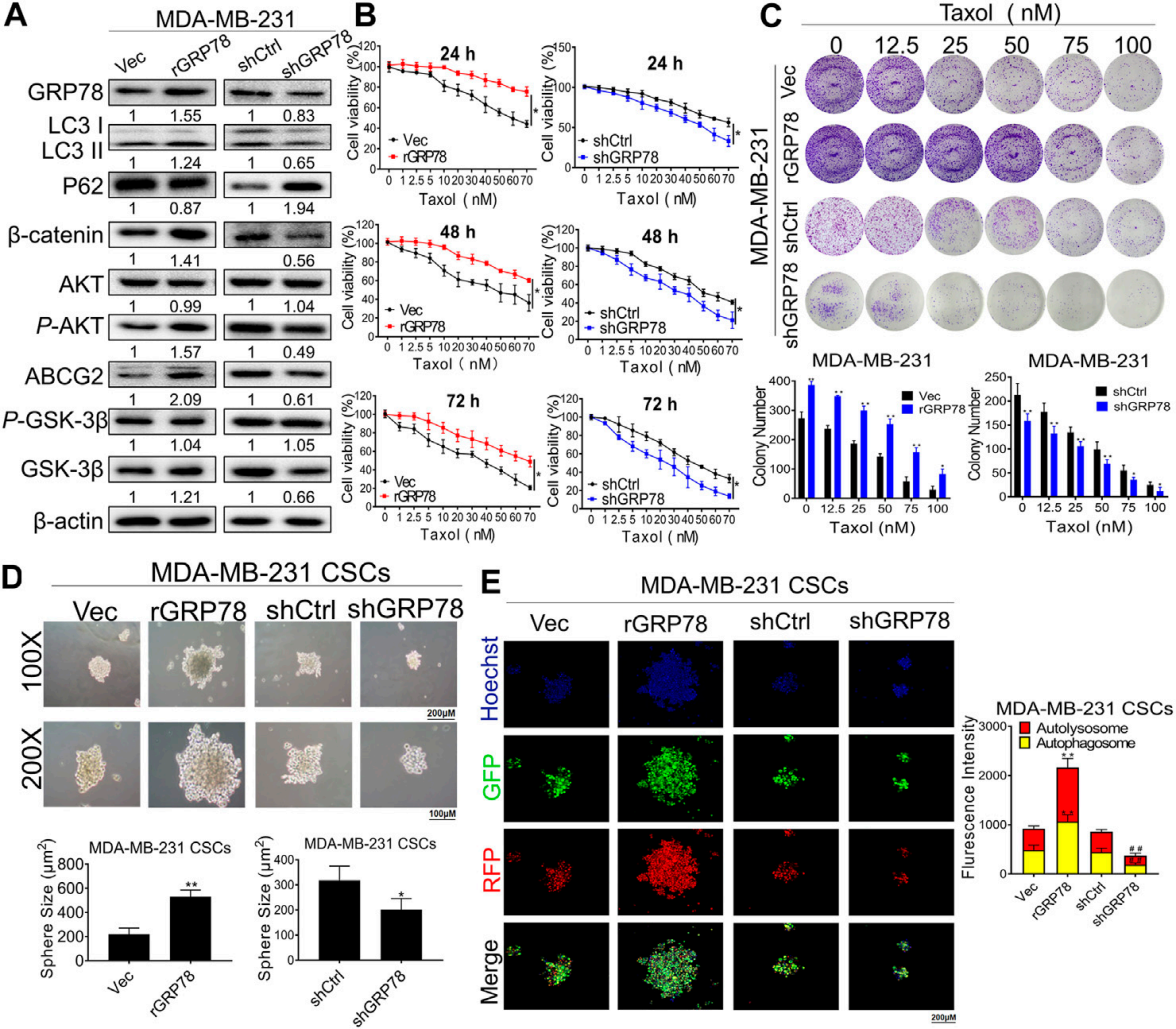
GRP78 decreases breast cancer chemosensitivity possibly via autophagy induction of breast CSCs. **(A)** Western blotting verified the expressions of GRP78, LC3, P62, β-catenin, ABCG2, GSK-3β, P-GSK-3β, AKT and P-AKT in MDA-MB-231 cells before or after the indicated transfection. **(B)** CCK8 assay detected the cell proliferation in GRP78^high^ and GRP78^low^ MDA-MB-231 cells with or without taxol administration. All values represent the means ± SD (*n* = 3, *p < 0.05, **p < 0.01 vs. Vec group; #*p* < 0.05, ##*p* < 0.01 vs. shCtrl group). **(C)** Colony formation assay was performed to evaluate the long-term inhibitory effects of ADQ on GRP78^high^ and GRP78^low^ MDA-MB-231 cells. All values represent the means ± SD (*n* = 3, *p < 0.05, **p < 0.01 vs. Control group). **(D)** Sphere-forming assay in MDA-MB-231 CSCs before or after the indicated transfection. All values represent the means ± SD (*n* = 3, *p < 0.05, **p < 0.01 vs. Control group). **(E)** Fluorescence photographs of autophagic flux transfected with an LC3-GFP-mRFP reporter in GRP78^high^ and GRP78^low^ MDA-MB-231 cells. All values represent the means ± SD (*n* = 3, *p < 0.05, **p < 0.01 vs Vec group; #*p* < 0.05, ##*p* < 0.01 vs. shCtrl group).

To better show a whole CSC sphere transfected with the mRFP-GFP-LC3 reporter, representative confocal images were selected under a low magnification (scale bar: 200 μm) in the original article. Therefore, a brief description should be added to the end of **Immunofluorescence Analysis**, indicating that “The mammospheres were dissociated into single-cell suspension for quantification of autophagosome/autolysosome under a higher magnification”.

The authors apologize for this error and state that this does not change the scientific conclusions of the article in any way. The original article has been updated.

