# Supplementary figures and images for: Autophagy Blockade by Ai Du Qing Formula Promotes Chemosensitivity of Breast Cancer Stem Cells Via GRP78/β-Catenin/ABCG2 Axis
Source: Front Pharmacol. 2021 Jun 3;12:659297. doi: 10.3389/fphar.2021.659297 (PMC8210424; doi:10.3389/fphar.2021.659297)

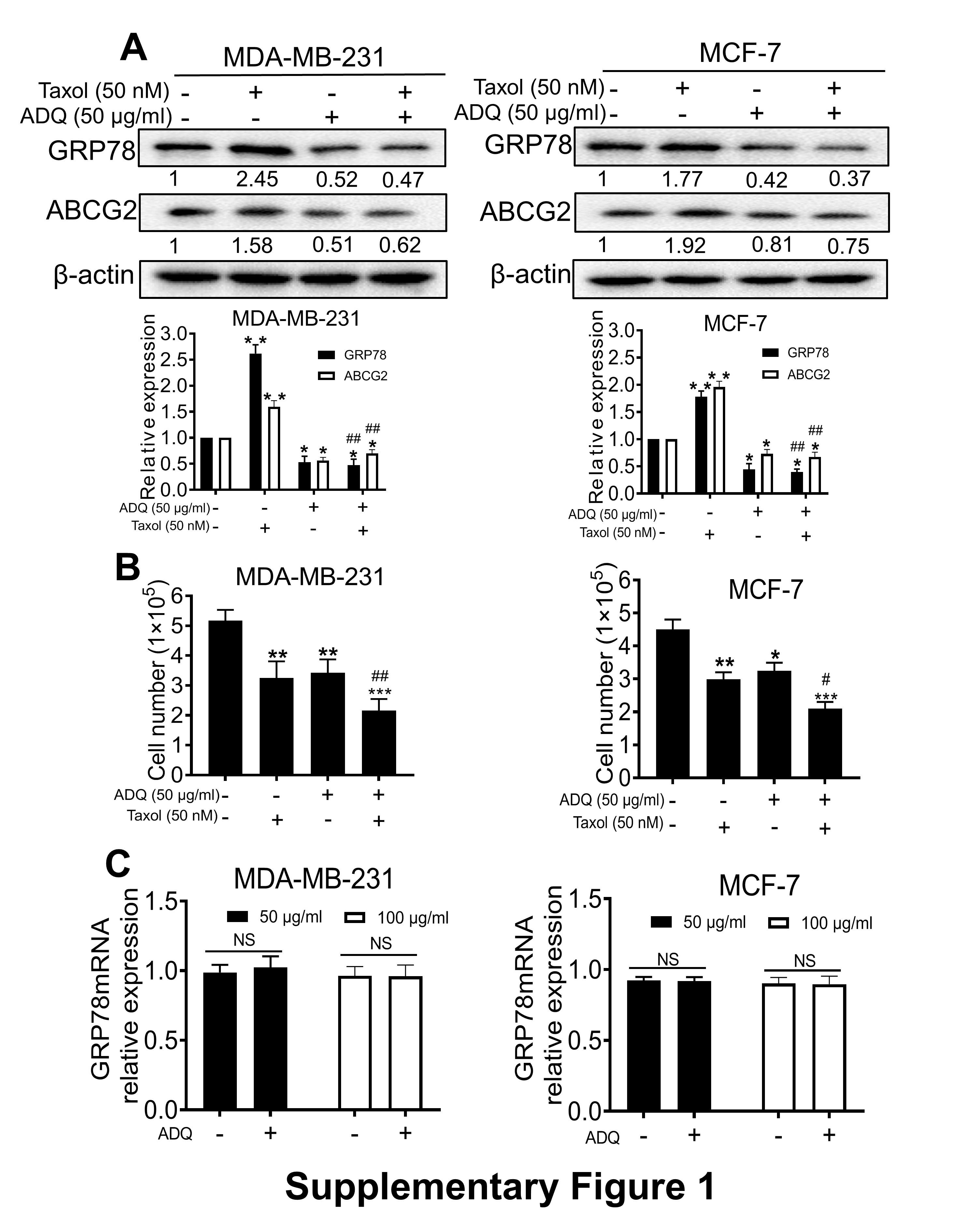

Supplement: Supplementary file 1 [file Image1.JPEG]
