# Supplementary material for: Autophagy Blockade by Ai Du Qing Formula Promotes Chemosensitivity of Breast Cancer Stem Cells Via GRP78/β-Catenin/ABCG2 Axis
Source: Front Pharmacol. 2021 Jun 3;12:659297. doi: 10.3389/fphar.2021.659297 (PMC8210424; doi:10.3389/fphar.2021.659297)
Supplement: Supplementary file 3 [file DataSheet1.PDF]

Figure 3 B

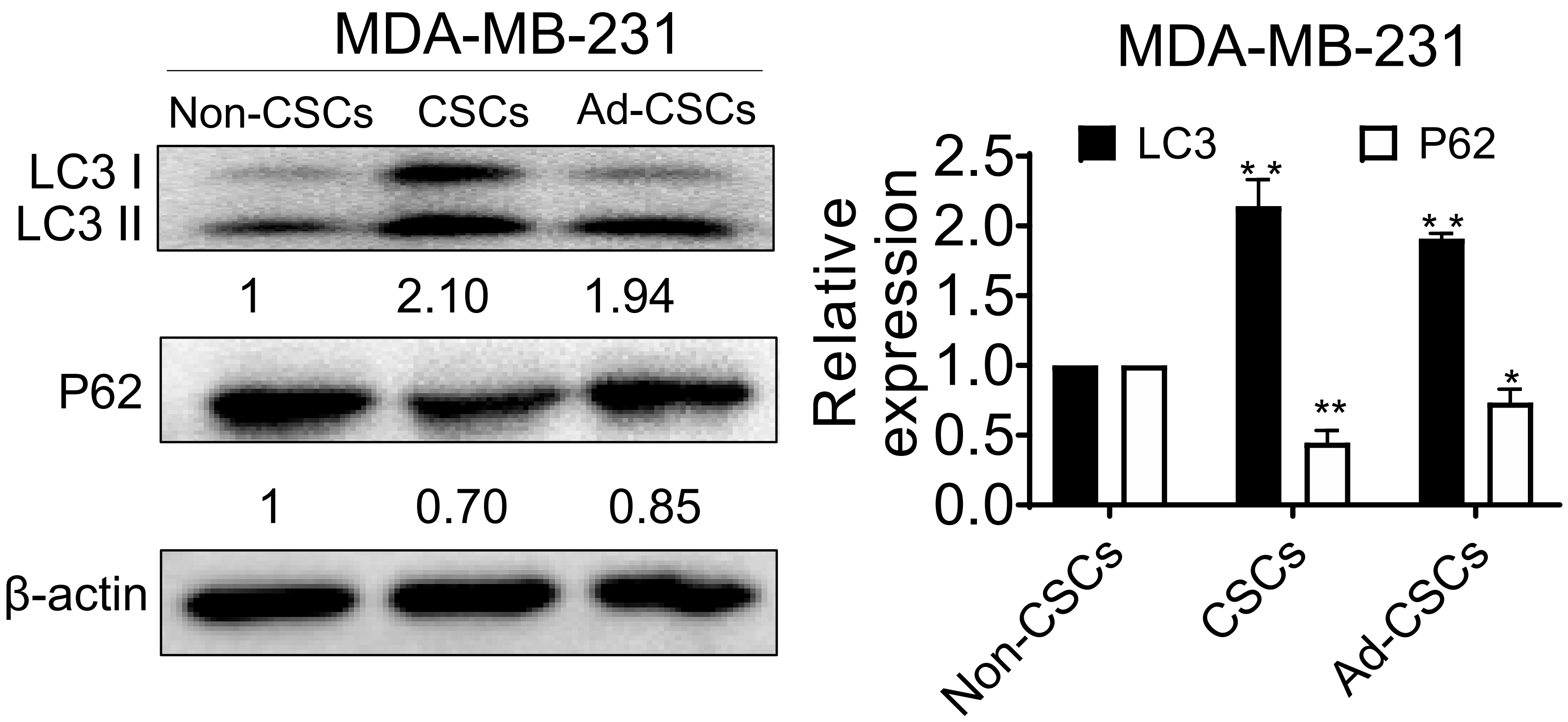
MDA-MB-231
Non-CSCs
CSCs
Ad-CSCs
LC3 I
LC3 II
1
2.10
1.94
P62
1
0.70
0.85
β-actin
MDA-MB-231
LC3
P62
Relative expression
2.5
2.0
1.5
1.0
0.5
0.0
**
**
**
*
Non-CSCs
CSCs
Ad-CSCs

Figure 3E

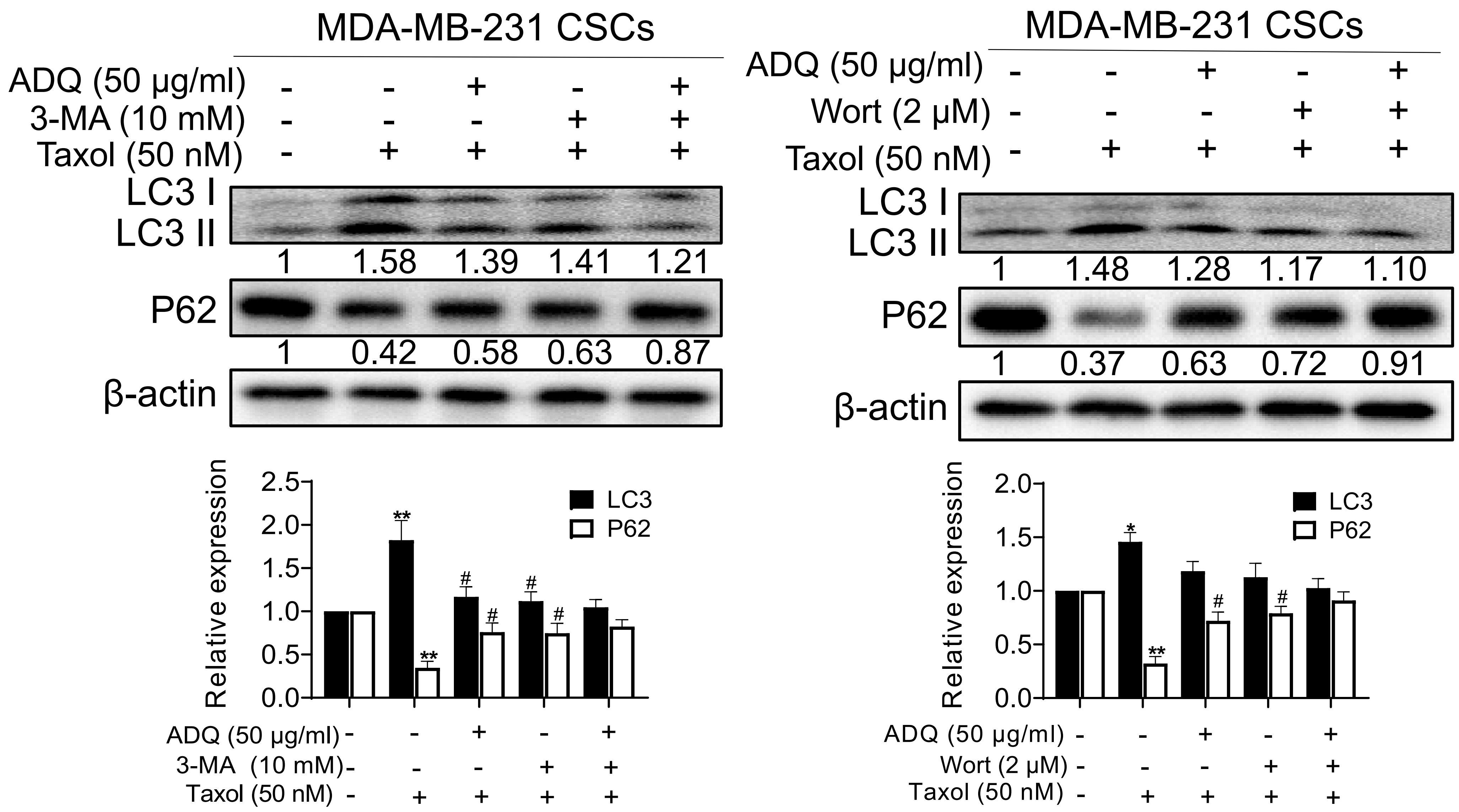
MDA-MB-231 CSCs
ADQ (50 μg/ml) - - + - +
3-MA (10 mM) - - - + +
Taxol (50 nM) - + + + +
LC3 I
LC3 II
1 1.58 1.39 1.41 1.21
P62
1 0.42 0.58 0.63 0.87
β-actin
Relative expression
2.5
2.0
1.5
1.0
0.5
0.0
LC3
P62
**
**
#
#
#
#
ADQ (50 μg/ml) - - + - +
3-MA (10 mM) - - - + +
Taxol (50 nM) - + + + +
MDA-MB-231 CSCs
ADQ (50 μg/ml) - - + - +
Wort (2 μM) - - - + +
Taxol (50 nM) - + + + +
LC3 I
LC3 II
1 1.48 1.28 1.17 1.10
P62
1 0.37 0.63 0.72 0.91
β-actin
Relative expression
2.0
1.5
1.0
0.5
0.0
LC3
P62
*
**
#
#
ADQ (50 μg/ml) - - + - +
Wort (2 μM) - - - + +
Taxol (50 nM) - + + + +

Figure 3E

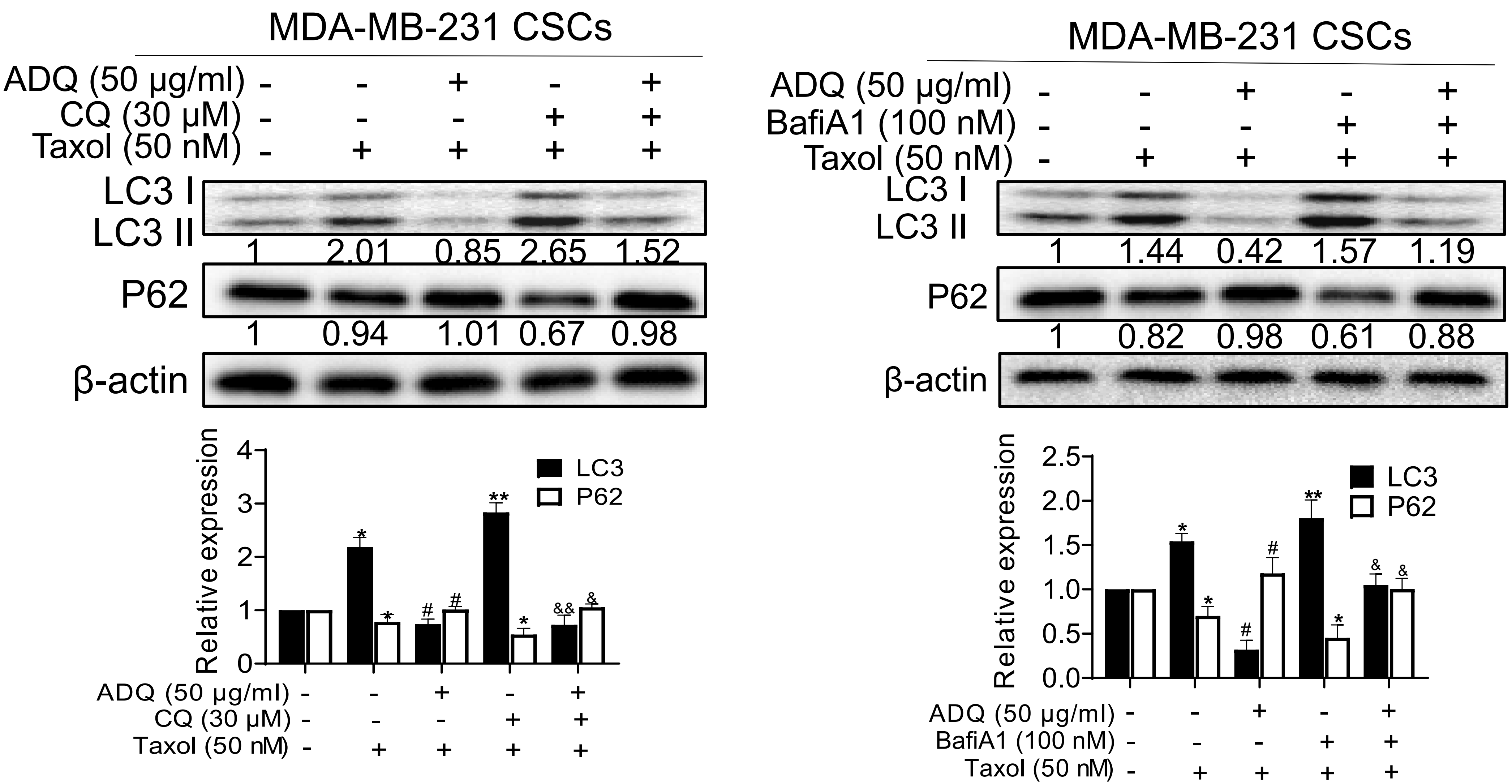

MDA-MB-231 CSCs
ADQ (50 μg/ml) - - + - +
CQ (30 μM) - - - + +
Taxol (50 nM) - + + + +
LC3 I
LC3 II
1 2.01 0.85 2.65 1.52
P62
1 0.94 1.01 0.67 0.98
β-actin
Relative expression
LC3
P62
MDA-MB-231 CSCs
ADQ (50 μg/ml) - - + - +
BafiA1 (100 nM) - - - + +
Taxol (50 nM) - + + + +
LC3 I
LC3 II
1 1.44 0.42 1.57 1.19
P62
1 0.82 0.98 0.61 0.88
β-actin
Relative expression
LC3
P62

Figure 4A

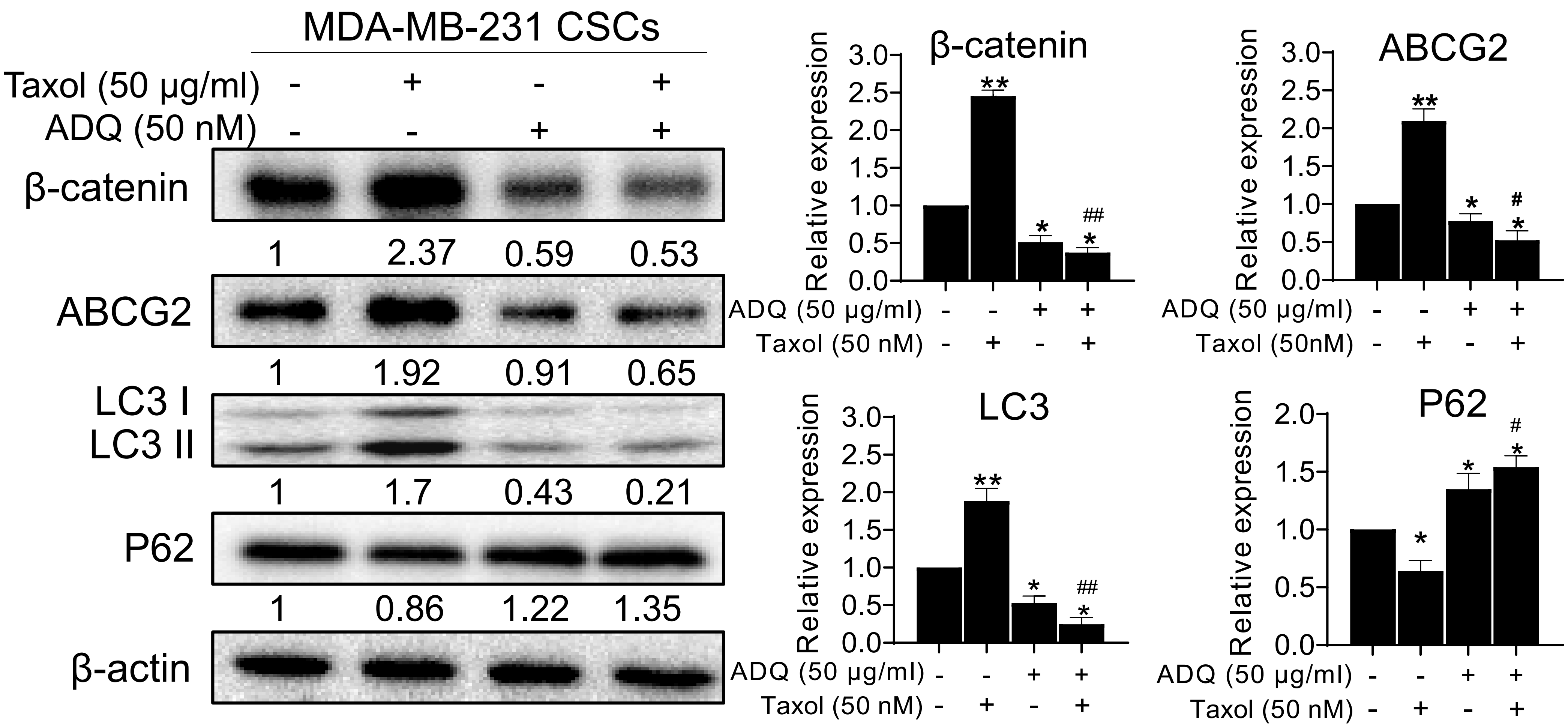

MDA-MB-231 CSCs
Taxol (50 μg/ml) - + - +
ADQ (50 nM) - - + +
β-catenin
1 2.37 0.59 0.53
ABCG2
1 1.92 0.91 0.65
LC3 I
LC3 II
1 1.7 0.43 0.21
P62
1 0.86 1.22 1.35
β-actin
β-catenin
ABCG2
LC3
P62
Relative expression
ADQ (50 μg/ml) - - + +
Taxol (50 nM) - + - +
Taxol (50nM) - + - +

Figure 4B

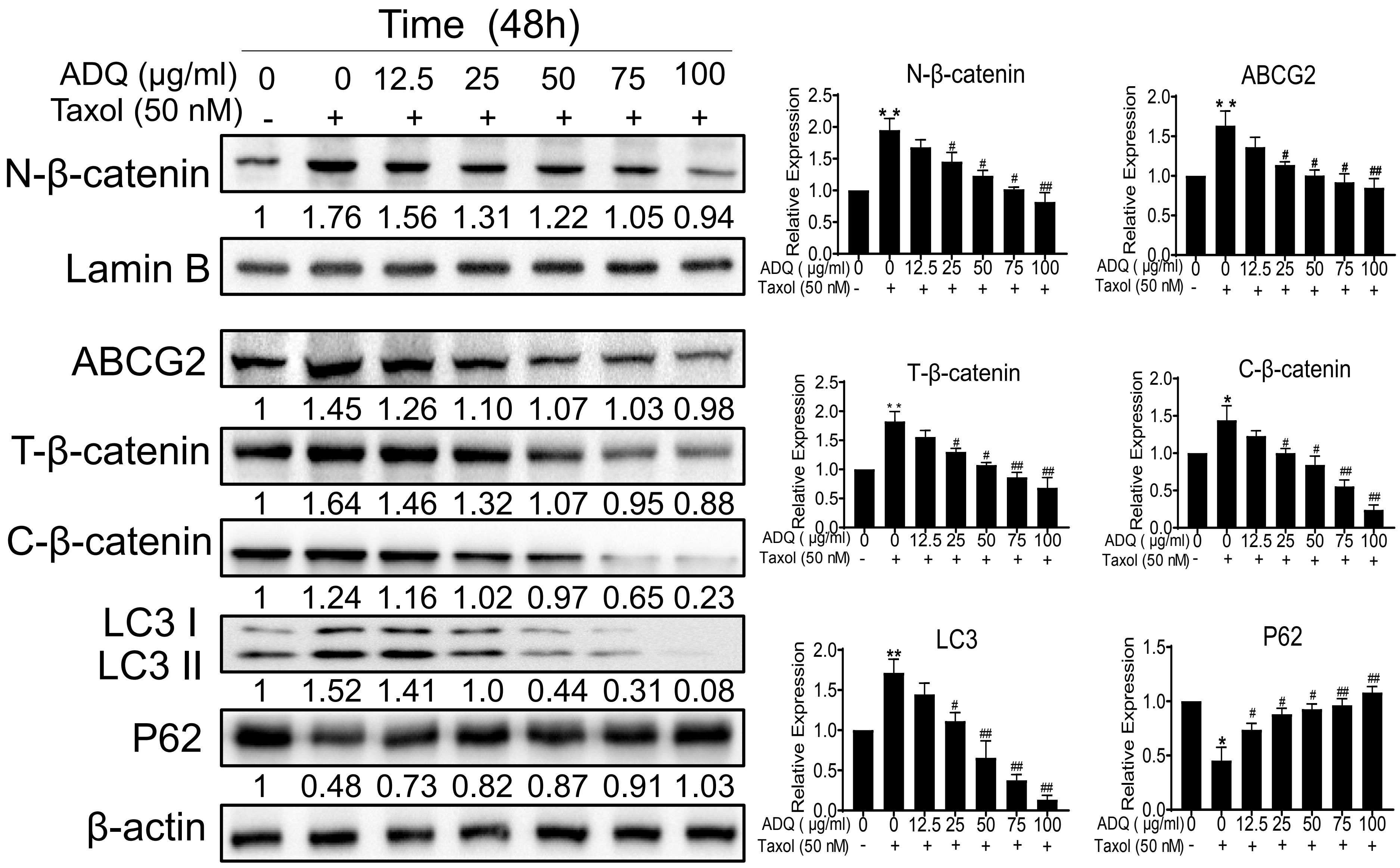

Time (48h)
ADQ (μg/ml) 0 0 12.5 25 50 75 100
Taxol (50 nM) - + + + + + +
N-β-catenin
1 1.76 1.56 1.31 1.22 1.05 0.94
Lamin B
ABCG2
1 1.45 1.26 1.10 1.07 1.03 0.98
T-β-catenin
1 1.64 1.46 1.32 1.07 0.95 0.88
C-β-catenin
1 1.24 1.16 1.02 0.97 0.65 0.23
LC3 I
LC3 II
1 1.52 1.41 1.0 0.44 0.31 0.08
P62
1 0.48 0.73 0.82 0.87 0.91 1.03
β-actin
N-β-catenin
ABCG2
T-β-catenin
C-β-catenin
LC3
P62
Relative Expression
ADQ (μg/ml) 0 0 12.5 25 50 75 100
Taxol (50 nM) - + + + + + +

Figure 4B

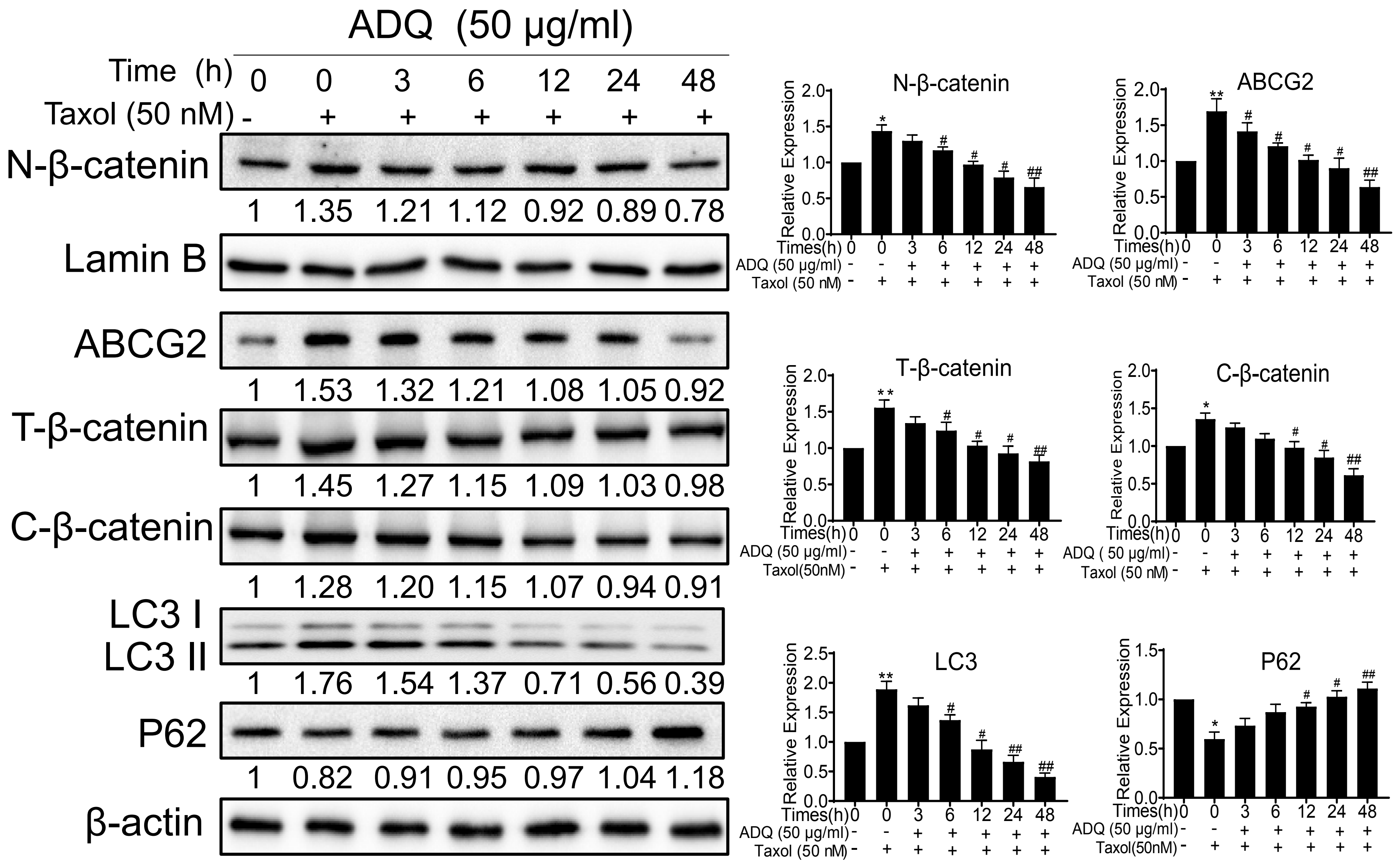
ADQ (50 μg/ml)
Time (h) 0 0 3 6 12 24 48
Taxol (50 nM) - + + + + + +
N-β-catenin
1 1.35 1.21 1.12 0.92 0.89 0.78
Lamin B
ABCG2
1 1.53 1.32 1.21 1.08 1.05 0.92
T-β-catenin
1 1.45 1.27 1.15 1.09 1.03 0.98
C-β-catenin
1 1.28 1.20 1.15 1.07 0.94 0.91
LC3 I
LC3 II
1 1.76 1.54 1.37 0.71 0.56 0.39
P62
1 0.82 0.91 0.95 0.97 1.04 1.18
β-actin
N-β-catenin
ABCG2
T-β-catenin
C-β-catenin
LC3
P62
Relative Expression
Times(h) 0 0 3 6 12 24 48
ADQ (50 μg/ml) - - + + + + +
Taxol (50 nM) - + + + + + +

Figure 4D

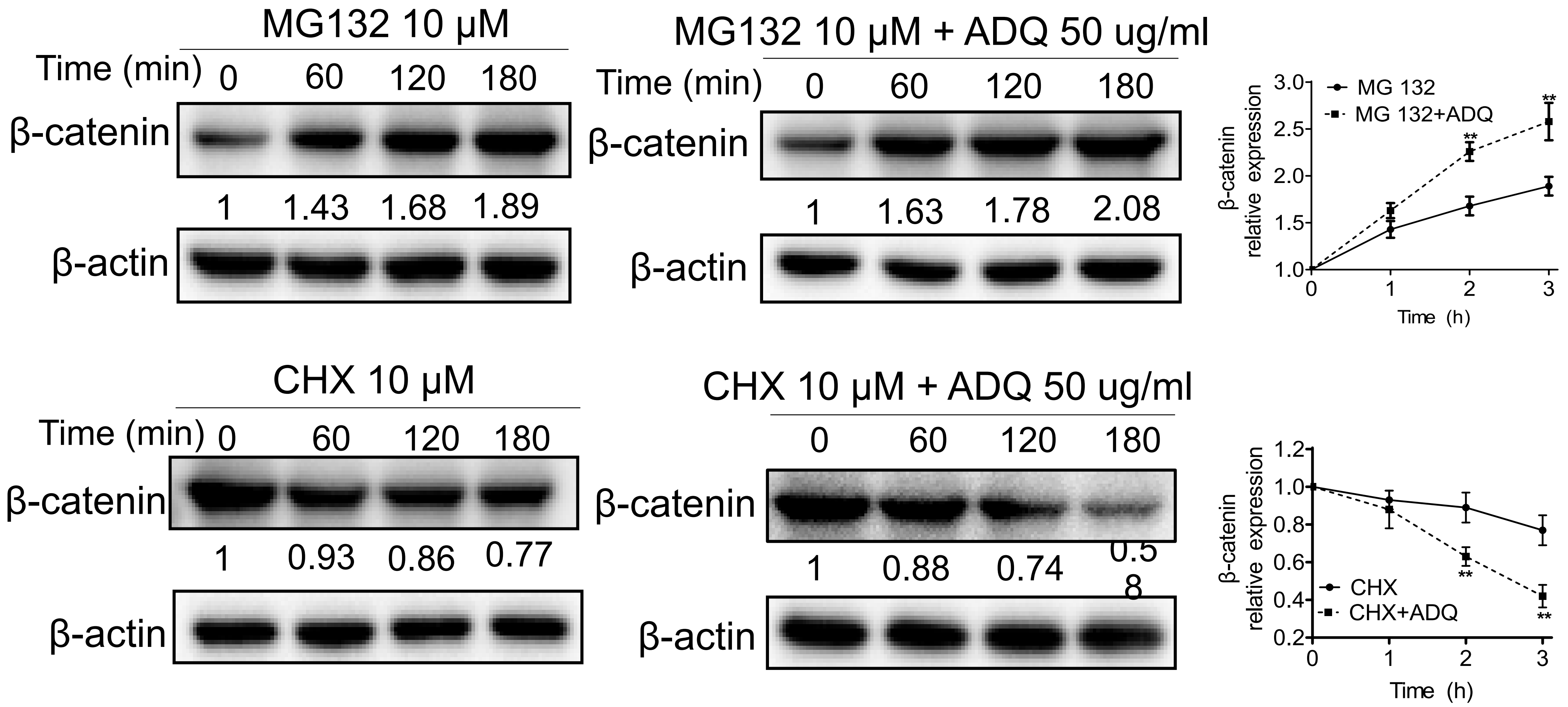

MG132 10 μM
Time (min) 0 60 120 180
β-catenin
1 1.43 1.68 1.89
β-actin
MG132 10 μM + ADQ 50 ug/ml
Time (min) 0 60 120 180
β-catenin
1 1.63 1.78 2.08
β-actin
MG 132
MG 132+ADQ
β-catenin relative expression
Time (h)
CHX 10 μM
Time (min) 0 60 120 180
β-catenin
1 0.93 0.86 0.77
β-actin
CHX 10 μM + ADQ 50 ug/ml
0 60 120 180
β-catenin
1 0.88 0.74 0.5 8
β-actin
CHX
CHX+ADQ
β-catenin relative expression
Time (h)

Figure 4E

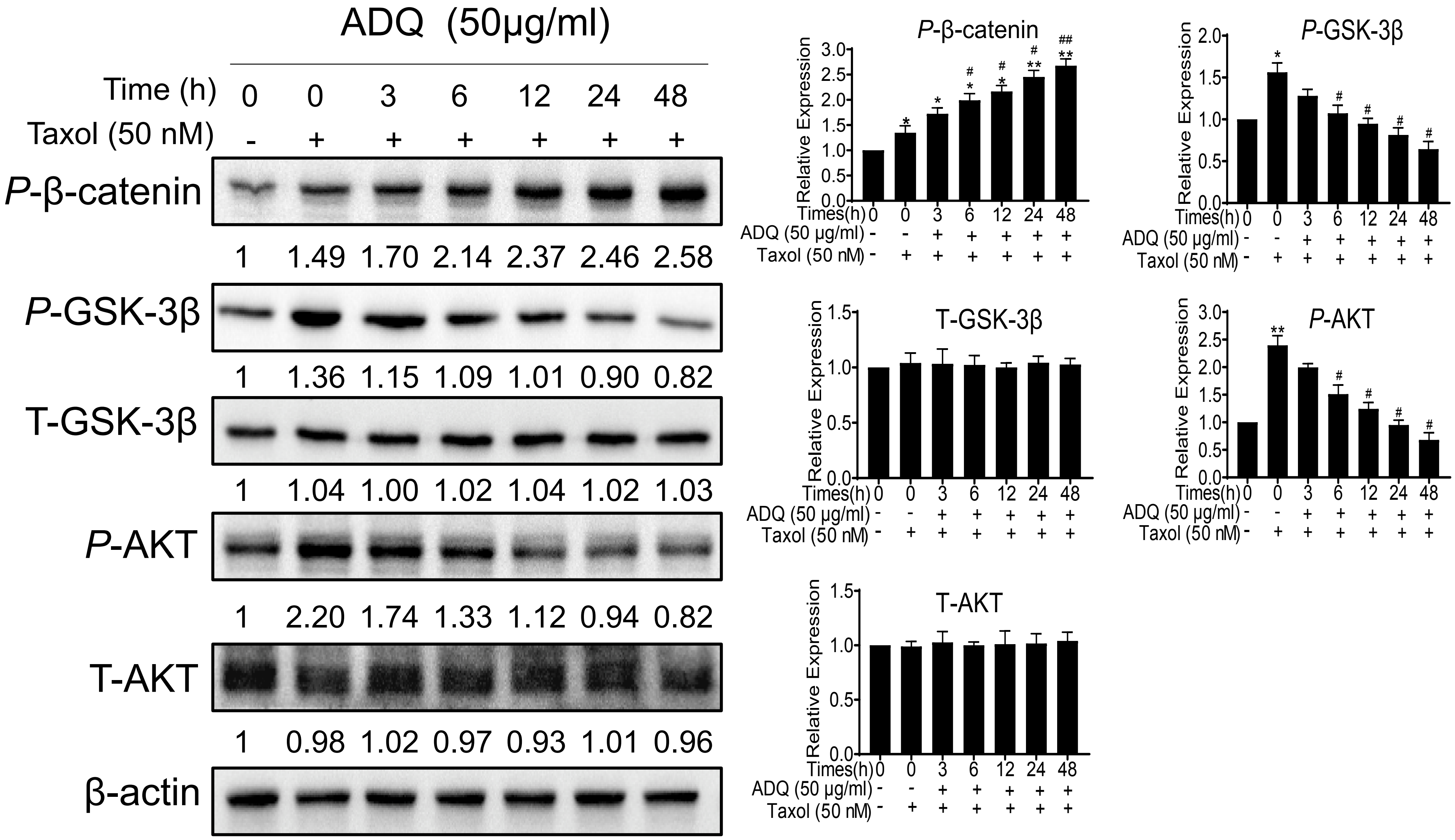
ADQ (50μg/ml)
Time (h) 0 0 3 6 12 24 48
Taxol (50 nM) - + + + + + +
P-β-catenin
1 1.49 1.70 2.14 2.37 2.46 2.58
P-GSK-3β
1 1.36 1.15 1.09 1.01 0.90 0.82
T-GSK-3β
1 1.04 1.00 1.02 1.04 1.02 1.03
P-AKT
1 2.20 1.74 1.33 1.12 0.94 0.82
T-AKT
1 0.98 1.02 0.97 0.93 1.01 0.96
β-actin
P-β-catenin
P-GSK-3β
T-GSK-3β
P-AKT
T-AKT
Relative Expression
Times(h) 0 0 3 6 12 24 48
ADQ (50 μg/ml) - - + + + + +
Taxol (50 nM) - + + + + + +

Figure 4E

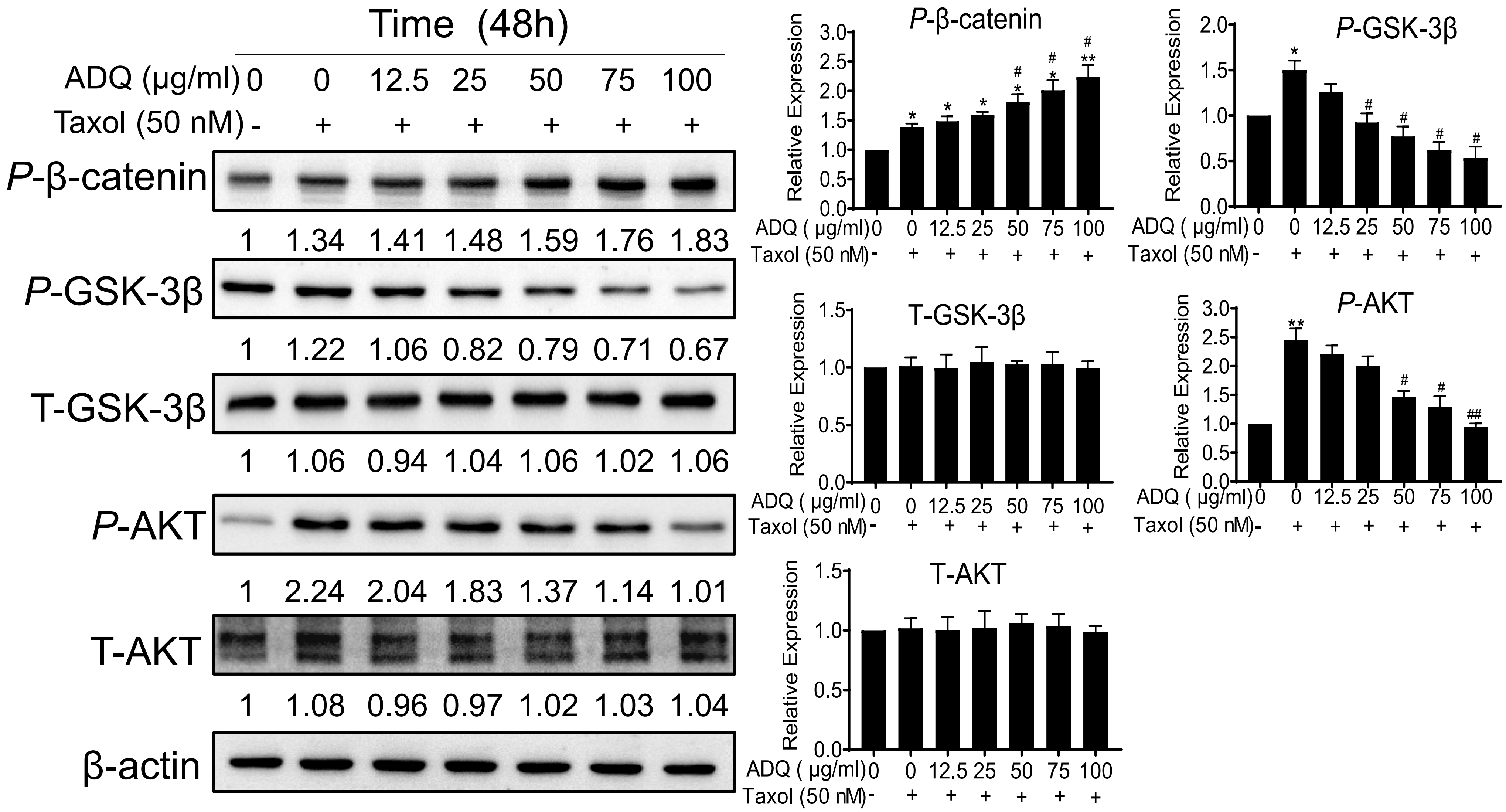
Time (48h)
ADQ (μg/ml) 0 0 12.5 25 50 75 100
Taxol (50 nM) - + + + + + +
P-β-catenin
1 1.34 1.41 1.48 1.59 1.76 1.83
P-GSK-3β
1 1.22 1.06 0.82 0.79 0.71 0.67
T-GSK-3β
1 1.06 0.94 1.04 1.06 1.02 1.06
P-AKT
1 2.24 2.04 1.83 1.37 1.14 1.01
T-AKT
1 1.08 0.96 0.97 1.02 1.03 1.04
β-actin
P-β-catenin
P-GSK-3β
T-GSK-3β
P-AKT
T-AKT
Relative Expression
ADQ (μg/ml) 0 0 12.5 25 50 75 100
Taxol (50 nM) - + + + + + +

Figure 4F

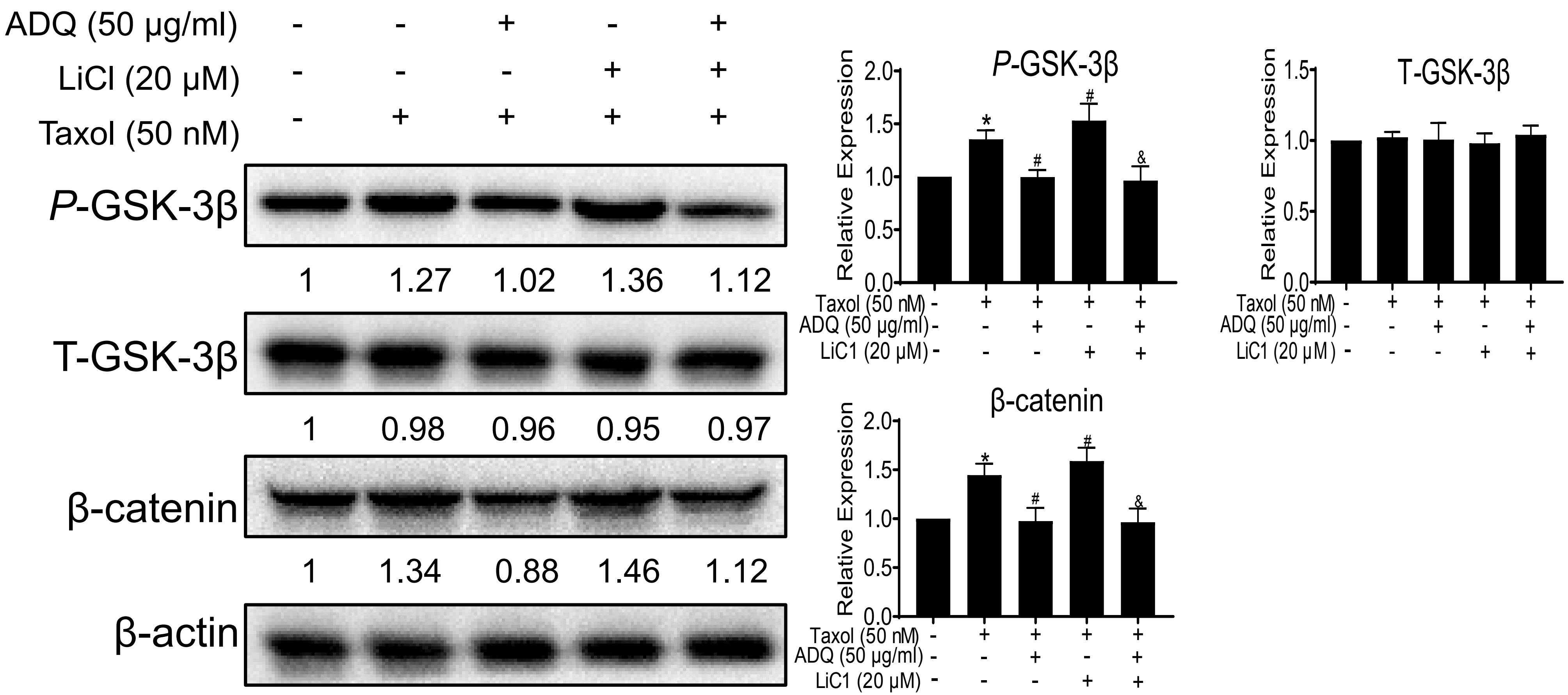
ADQ (50 μg/ml) - - + - +
LiCl (20 μM) - - - + +
Taxol (50 nM) - + + + +
P-GSK-3β
1 1.27 1.02 1.36 1.12
T-GSK-3β
1 0.98 0.96 0.95 0.97
β-catenin
1 1.34 0.88 1.46 1.12
β-actin
P-GSK-3β
T-GSK-3β
β-catenin
Relative Expression
Taxol (50 nM) - + + + +
ADQ (50 μg/ml) - - + - +
LiC1 (20 μM) - - - + +

Figure 4F

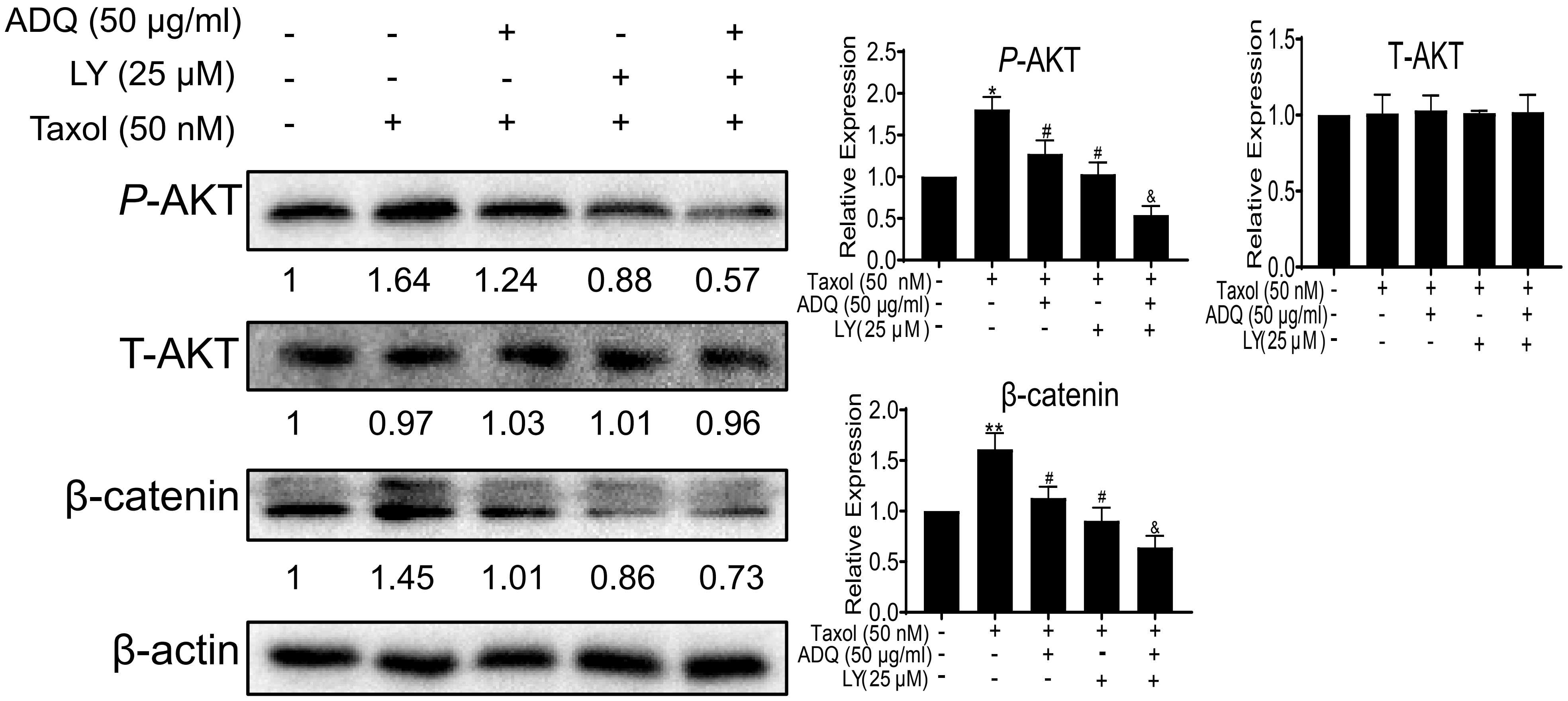
ADQ (50 μg/ml) - - + - +
LY (25 μM) - - - + +
Taxol (50 nM) - + + + +
P-AKT
1 1.64 1.24 0.88 0.57
T-AKT
1 0.97 1.03 1.01 0.96
β-catenin
1 1.45 1.01 0.86 0.73
β-actin
P-AKT
Relative Expression
T-AKT
β-catenin
Taxol (50 nM) - + + + +
ADQ (50 μg/ml) - - + - +
LY(25 μM) - - - + +

Figure 5A

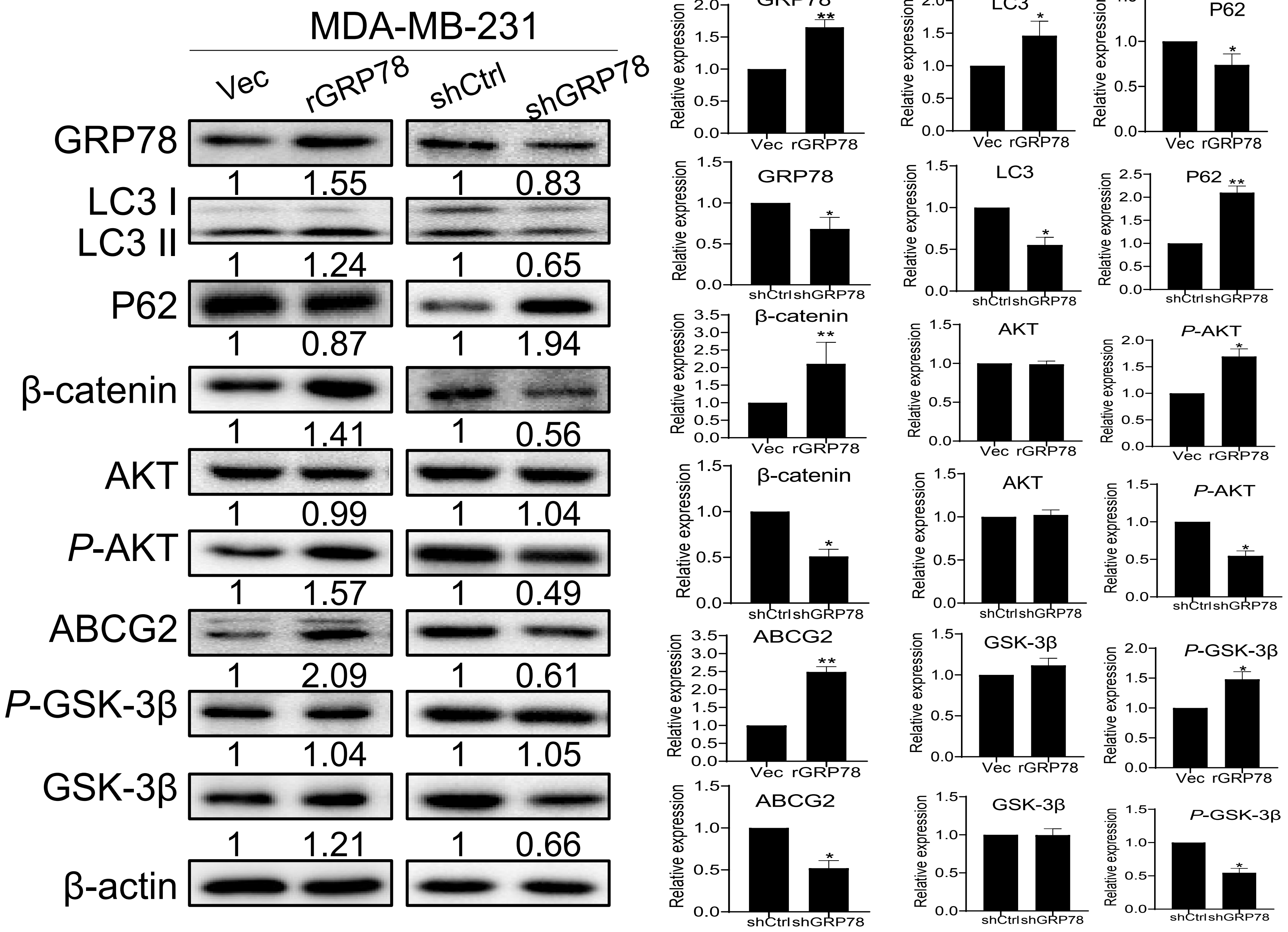
MDA-MB-231
Vec
rGRP78
shCtrl
shGRP78
GRP78
1 1.55 1 0.83
LC3 I
LC3 II
1 1.24 1 0.65
P62
1 0.87 1 1.94
β-catenin
1 1.41 1 0.56
AKT
1 0.99 1 1.04
P-AKT
1 1.57 1 0.49
ABCG2
1 2.09 1 0.61
P-GSK-3β
1 1.04 1 1.05
GSK-3β
1 1.21 1 0.66
β-actin
Relative expression
GRP78
LC3
P62
β-catenin
AKT
P-AKT
ABCG2
GSK-3β
P-GSK-3β
Vec rGRP78
shCtrl shGRP78
**
*

Figure 6A

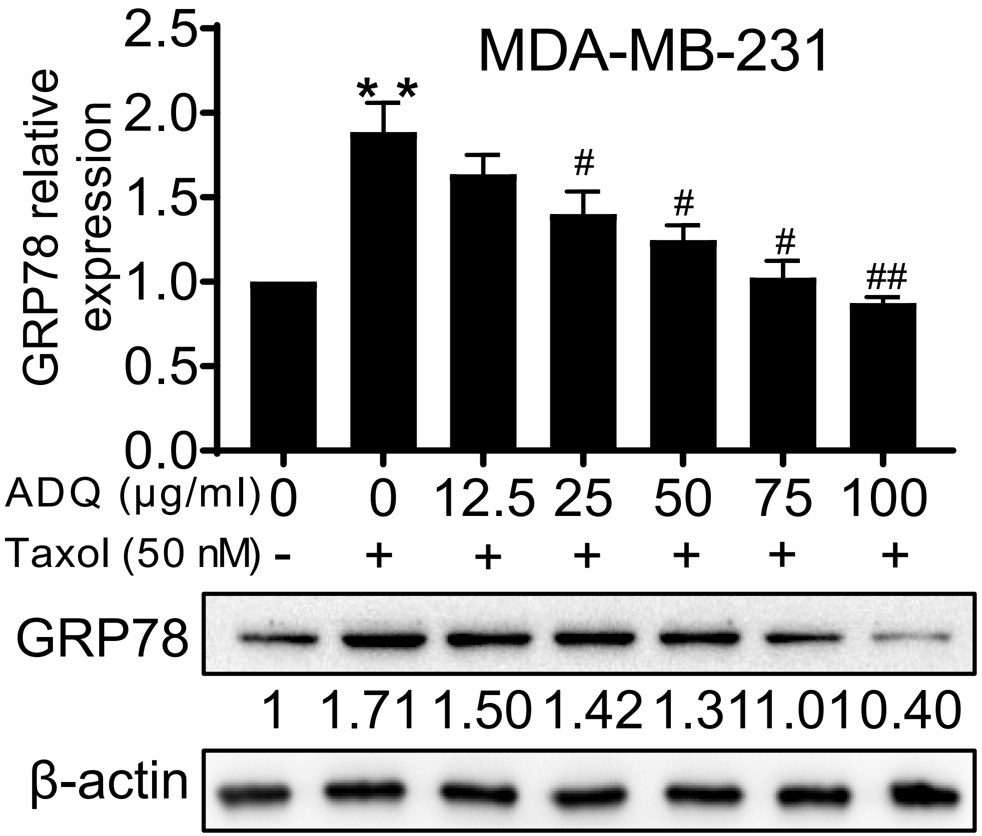

MDA-MB-231
GRP78 relative expression
2.5
2.0
1.5
1.0
0.5
0.0
* *
#
#
#
##
ADQ (μg/ml) 0 0 12.5 25 50 75 100
Taxol (50 nM) - + + + + + +
GRP78
1 1.71 1.50 1.42 1.31 1.01 0.40
β-actin
MCF-7
GRP78 relative Expression
2.5
2.0
1.5
1.0
0.5
0.0
* *
#
#
#
ADQ (μg/ml) 0 0 12.5 25 50 75 100
Taxol (50 nM) - + + + + + +
GRP78
1 2.31 2.11 2.03 1.75 1.38 0.62
β-actin

Figure 6C

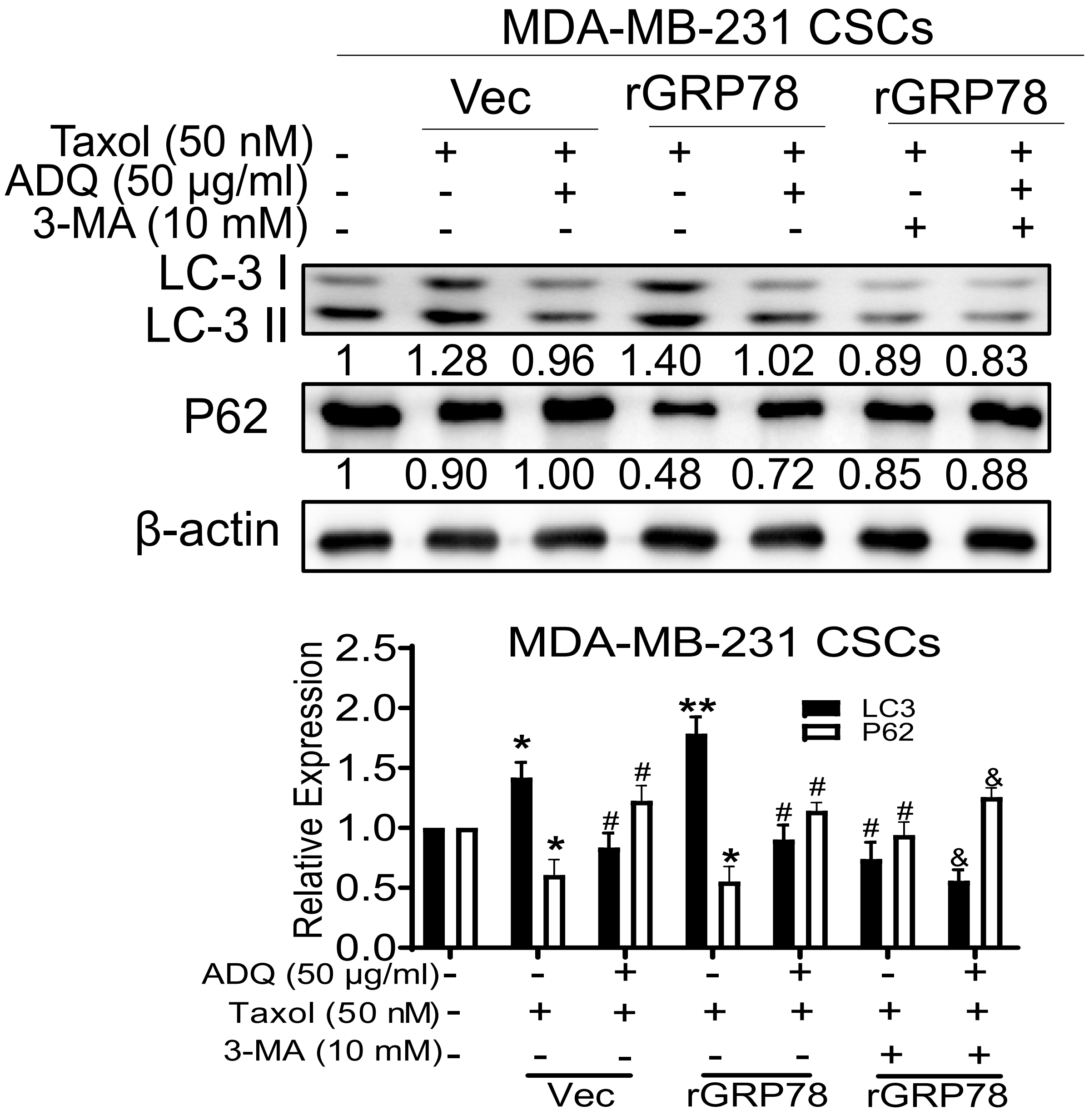

MDA-MB-231 CSCs
Vec
rGRP78
rGRP78
Taxol (50 nM)
ADQ (50 µg/ml)
3-MA (10 mM)
LC-3 I
LC-3 II
1 1.28 0.96 1.40 1.02 0.89 0.83
P62
1 0.90 1.00 0.48 0.72 0.85 0.88
β-actin
MDA-MB-231 CSCs
Relative Expression
LC3
P62
ADQ (50 µg/ml)
Taxol (50 nM)
3-MA (10 mM)
Vec
rGRP78
rGRP78

Figure 6E

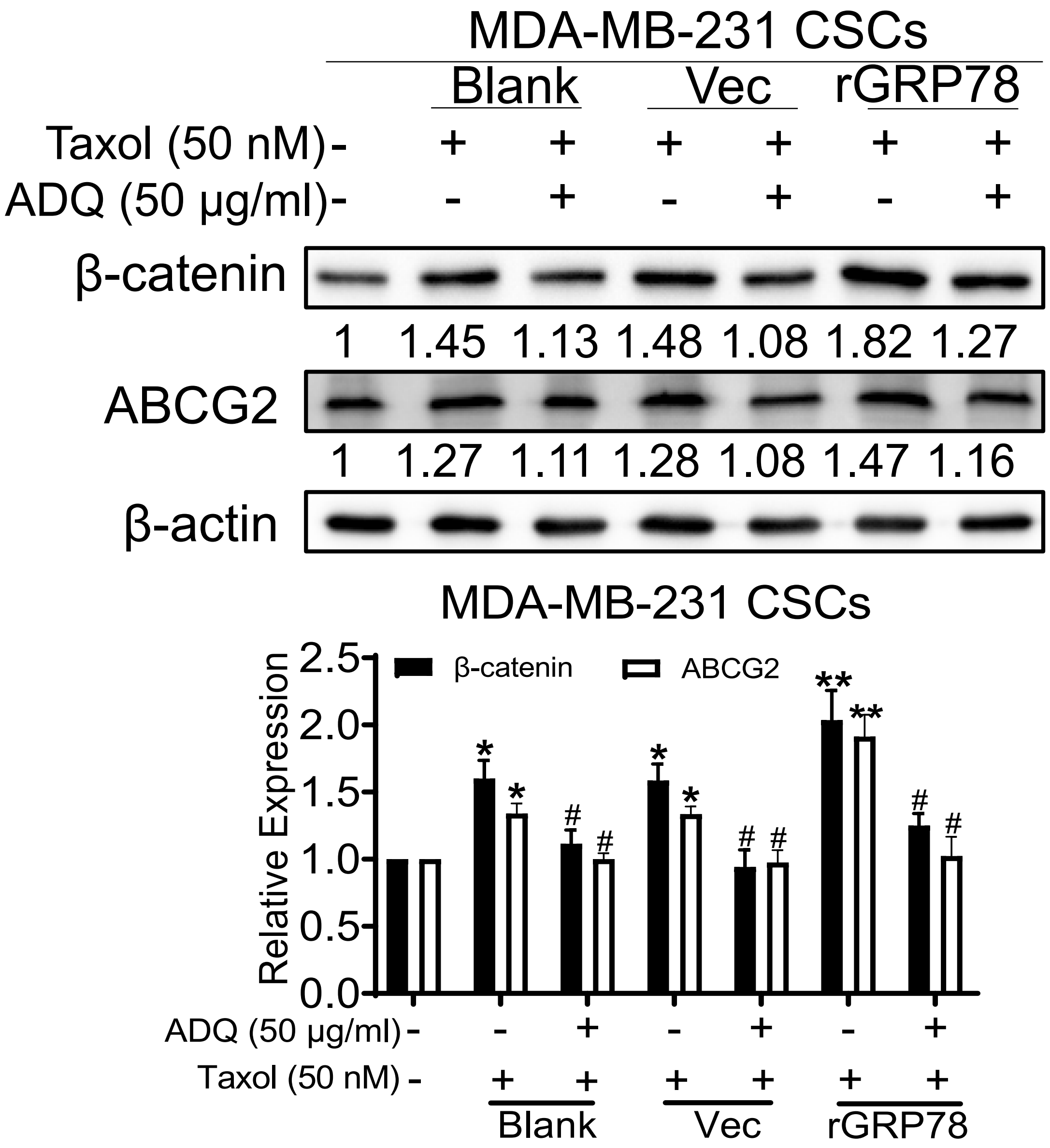
MDA-MB-231 CSCs
Blank
Vec
rGRP78
Taxol (50 nM) - + + + + + +
ADQ (50 μg/ml) - - + - + - +
β-catenin
1 1.45 1.13 1.48 1.08 1.82 1.27
ABCG2
1 1.27 1.11 1.28 1.08 1.47 1.16
β-actin
MDA-MB-231 CSCs
Relative Expression
β-catenin
ABCG2
2.5
2.0
1.5
1.0
0.5
0.0
ADQ (50 μg/ml) - - + - + - +
Taxol (50 nM) - + + + + + +
Blank
Vec
rGRP78

Figure 6E

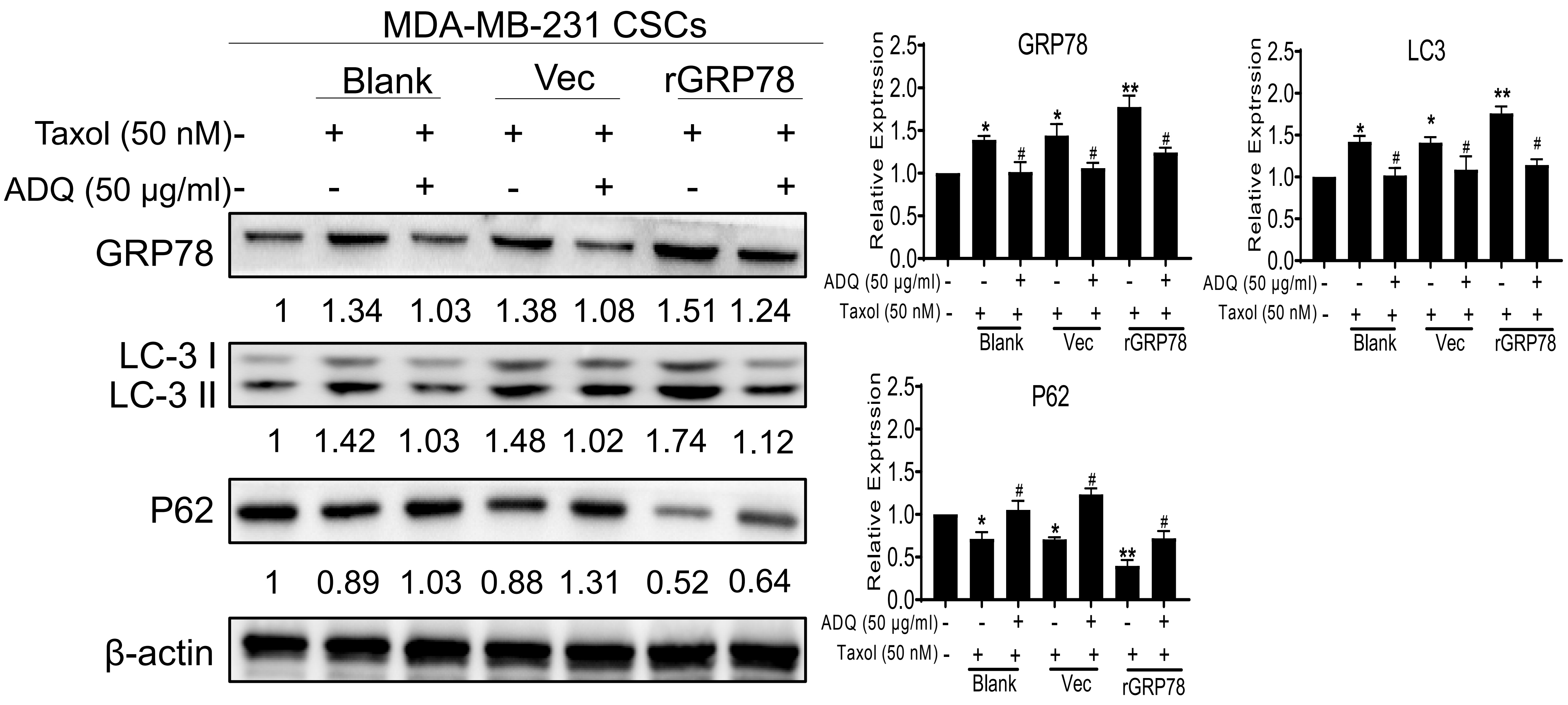
MDA-MB-231 CSCs
Blank
Vec
rGRP78
Taxol (50 nM)- + + + + + +
ADQ (50 μg/ml)- - + - + - +
GRP78
1 1.34 1.03 1.38 1.08 1.51 1.24
LC-3 I
LC-3 II
1 1.42 1.03 1.48 1.02 1.74 1.12
P62
1 0.89 1.03 0.88 1.31 0.52 0.64
β-actin
GRP78
LC3
P62
Relative Exptrssion
0.0
0.5
1.0
1.5
2.0
2.5
ADQ (50 μg/ml) - - + - + - +
Taxol (50 nM) - + + + + + +
Blank
Vec
rGRP78

Figure 6H

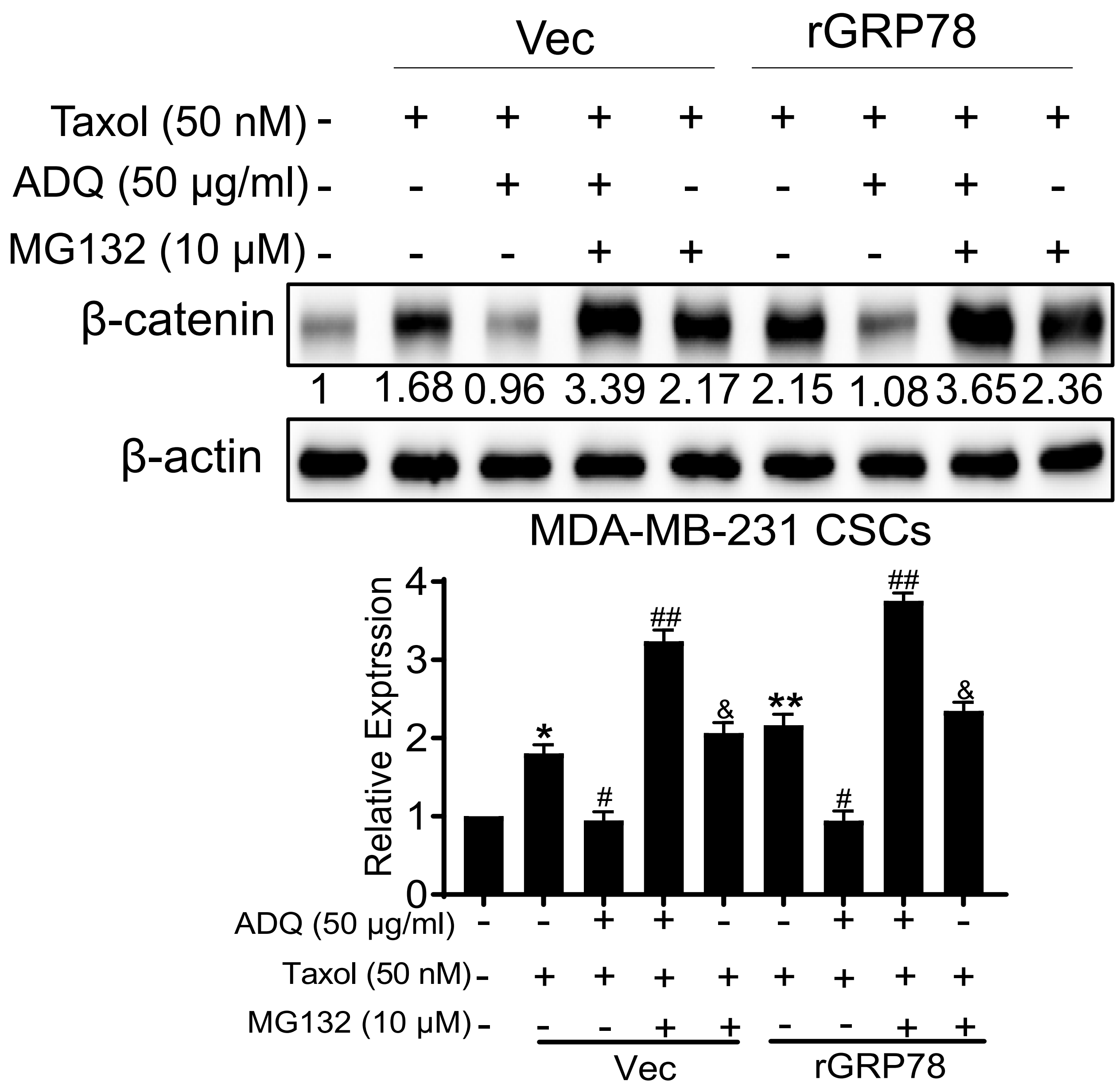
Vec
rGRP78
Taxol (50 nM) - + + + + + + + +
ADQ (50 μg/ml) - - + + - - + + -
MG132 (10 μM) - - - + + - - + +
β-catenin
1 1.68 0.96 3.39 2.17 2.15 1.08 3.65 2.36
β-actin
MDA-MB-231 CSCs
Relative Exptrssion
0
1
2
3
4
*
#
##
&
**
#
##
&
ADQ (50 μg/ml) - - + + - - + + -
Taxol (50 nM) - + + + + + + + +
MG132 (10 μM) - - - + + - - + +
Vec
rGRP78
