# Supplementary material for: Autophagy Blockade by Ai Du Qing Formula Promotes Chemosensitivity of Breast Cancer Stem Cells Via GRP78/β-Catenin/ABCG2 Axis
Source: Front Pharmacol. 2021 Jun 3;12:659297. doi: 10.3389/fphar.2021.659297 (PMC8210424; doi:10.3389/fphar.2021.659297)
Supplement: Supplementary file 4 [file DataSheet2.docx]

## Supplementary Figures

**
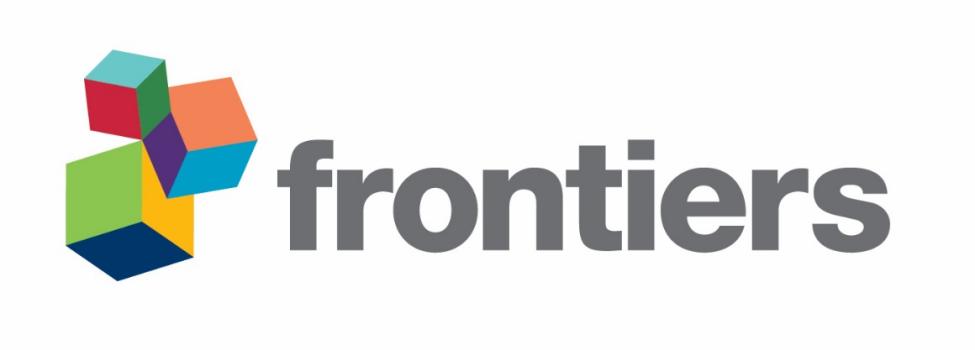
**

**Supplementary Figure 1.** **The suppression of GRP78 by ADQ may be posttranslational rather than transcriptional.** **(A)** Western blotting analysis showed that ADQ not only abolished the expressions of GRP78 and ABCG2, but also offset the facilitation effect of taxol on their expressions. All values represent the means ± SD (n=3, ^*^*P*<0.05, ^**^*P*<0.01 *vs.* Control group; ^#^*P*<0.05, ^##^*P*<0.01 *vs.* Taxol group). **(B)** The cell counting assay demonstrated that either ADQ or taxol alone could greatly limit cell growth in MDA-MB-231 cells and MCF-7 cells, while the combination group exerted the most significant reduction in comparison with a single group. All values represent the means ± SD (n=3, ^*^*P*<0.05, ^**^*P*<0.01 *vs.* Control group; ^#^*P*<0.05, ^##^*P*<0.01 *vs.* Taxol group). (C) PCR analysis demonstrated that ADQ administration led to little influence on GRP78 mRNA level in MDA-MB-231 cells and MCF-7 cells.

**Supplementary Figure 2. The steps of flow cytometry gating in Figure 7E. (A)** Gating procedure of DEAB^+^ group. **(B)** Gating procedure of DEAB^-^ group. FITC-A presents the ALDH^+^ population**.**

**Supplementary Figure 3. All western blotting results and statistical results.**
